# High-Throughput Identification of Crystalline Natural Products from Crude Extracts Enabled by Microarray Technology and microED

**DOI:** 10.1021/acscentsci.3c01365

**Published:** 2023-12-20

**Authors:** David A. Delgadillo, Jessica E. Burch, Lee Joon Kim, Lygia S. de Moraes, Kanji Niwa, Jason Williams, Melody J. Tang, Vincent G. Lavallo, Bhuwan Khatri Chhetri, Christopher G. Jones, Isabel Hernandez Rodriguez, Joshua A. Signore, Lewis Marquez, Riya Bhanushali, Sunmin Woo, Julia Kubanek, Cassandra Quave, Yi Tang, Hosea M. Nelson

**Affiliations:** † Division of Chemistry and Chemical Engineering, California Institute of Technology, Pasadena, California 91125, United States; ^‡^Department of Chemistry and Biochemistry, and ^§^Department of Chemical and Biomolecular Engineering, University of California, Los Angeles, Los Angeles, California 90095, United States; ∥ School of Biological Sciences, School of Chemistry and Biochemistry, and Neuroscience Program, Georgia Institute of Technology, Atlanta, Georgia 30332, United States; ⊥ Molecular and Systems Pharmacology, Laney Graduate School, Emory University, Atlanta, Georgia 30322, United States; ¶ Center for the Study of Human Health, Emory University, Atlanta, Georgia 30322, United States; # Department of Dermatology, Emory University School of Medicine, Atlanta, Georgia 30322, United States

## Abstract

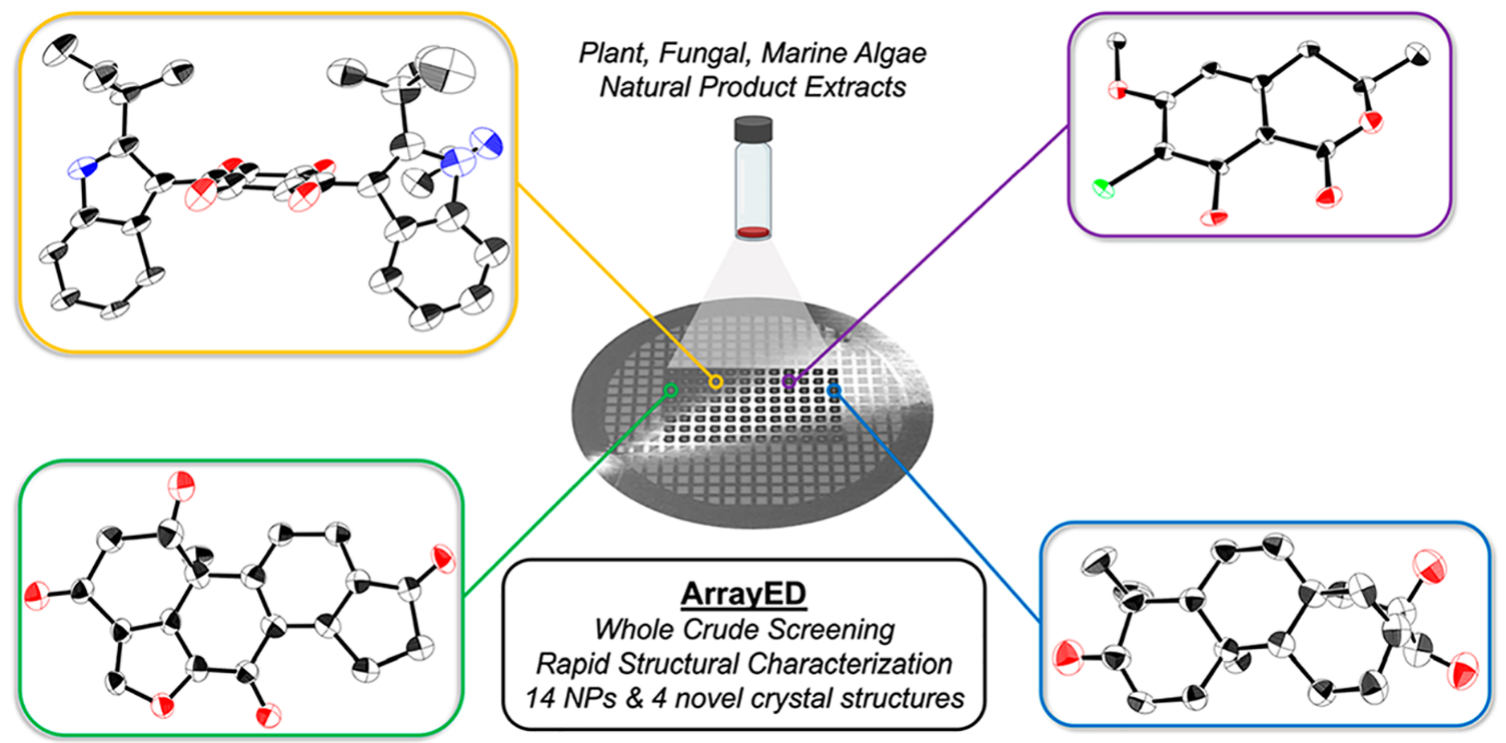

The structural determination of natural products (NPs) can be arduous because of sample heterogeneity. This often demands iterative purification processes and characterization of complex molecules that may be available only in miniscule quantities. Microcrystal electron diffraction (microED) has recently shown promise as a method to solve crystal structures of NPs from nanogram quantities of analyte. However, its implementation in NP discovery remains hampered by sample throughput and purity requirements, akin to traditional NP-discovery workflows. In the methods described herein, we leverage the resolving power of transmission electron microscopy (TEM) and the miniaturization capabilities of deoxyribonucleic acid (DNA) microarray technology to address these challenges through the establishment of an NP screening platform, array electron diffraction (ArrayED). In this workflow, an array of high-performance liquid chromatography (HPLC) fractions taken from crude extracts was deposited onto TEM grids in picoliter-sized droplets. This multiplexing of analytes on TEM grids enables 1200 or more unique samples to be simultaneously inserted into a TEM instrument equipped with an autoloader. Selected area electron diffraction analysis of these microarrayed grids allows for the rapid identification of crystalline metabolites. In this study, ArrayED enabled structural characterization of 14 natural products, including four novel crystal structures and two novel polymorphs, from 20 crude extracts. Moreover, we identify several chemical species that would not be detected by standard mass spectrometry (MS) or ultraviolet–visible (UV/vis) spectroscopy and crystal forms that would not be characterized using traditional methods.

## Abstract 2

ArrayED provides a high-throughput workflow by joining microarray technology with microcrystalline electron diffraction to rapidly screen time-resolved crude extracts and identify natural products from complex mixtures.

## Introduction

**Figure 1:**
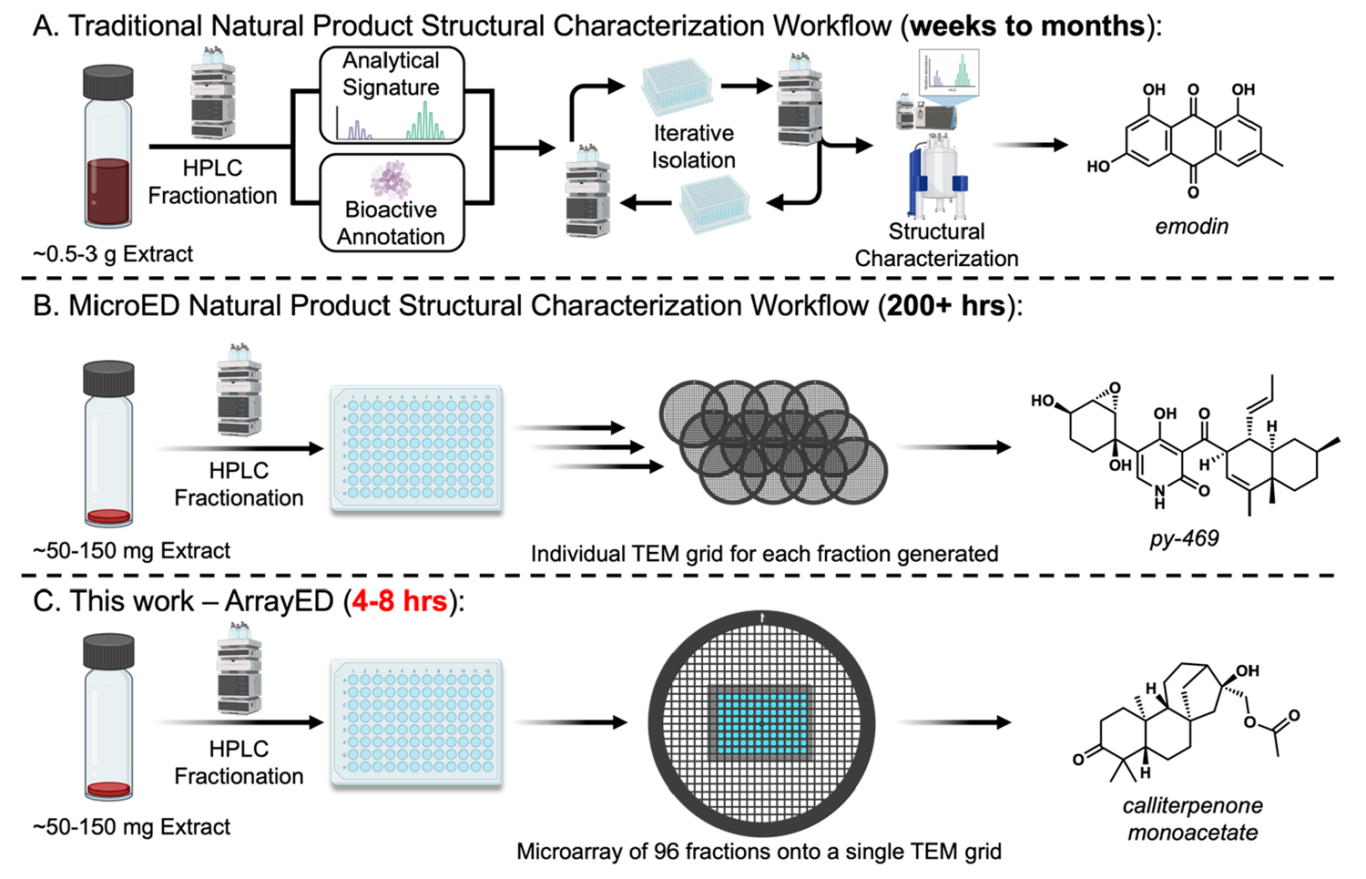
(A) Natural product isolation and characterization workflows. Traditional NP structural characterization involving iterative HPLC fractionation and purification followed by analytical interrogation. (B) Single-sample evaluation of NPs via microED. (C) High-throughput microarrayed TEM grid for microED structural elucidation.

Small molecules produced by living organisms, termed natural products (NPs), have been studied for centuries because of their fascinating structural diversity and potent biological activities. While NPs form the basis for many modern therapeutics,^1−3^ only a small fraction of the world’s NP space has been explored. Thus, the discovery of new chemical matter within this space will undoubtedly provide access to new therapeutic leads.^4^ The challenge in discovering new NPs is driven by difficulties in extraction, isolation, and purification of a single compound of interest from raw organic matter containing hundreds or even thousands of diverse small molecules.^5,6^ This process is further mired by the fact that natural products can be quite structurally complex, thereby necessitating intensive characterization on the basis of multiple analytical techniques, including 1D and 2D NMR (^1^H, ^13^C, etc.), FTIR, and MS (Figure 1a).^7−10^ Therefore, the development of high-throughput (HT) techniques that provide unambiguous identification of NPs would alleviate these challenges and profoundly impact current drug discovery efforts.

Single-crystal X-ray diffraction (SCXRD) remains the gold standard in small molecule characterization because structures can be determined unambiguously without prior knowledge or inference. However, SCXRD is often limited in practice by the need for large quantities (typically >1 mg) of purified material. The electron crystallography technique, microED or 3D ED, has recently gained interest among chemists as a powerful crystallographic method for the structural characterization of small molecules from nanograms of source material. Since electrons have charge and mass, they interact with matter more strongly than X-rays, and thus, electron diffraction experiments can yield data sufficient for structural determination from crystals less than one-billionth the size of crystals used for SCXRD (Figure 1b).^11−18^

While crystallization screens for SCXRD can be quickly triaged using an optical microscope, the formation of micro- and nanocrystals for microED experiments are most reliably evaluated under the high magnification of a transmission electron microscopy (TEM).^19,20^ However, screening for micro- and nanocrystals utilizing a TEM can be arduous because the deposition of a single sample on a TEM grid, followed by insertion and retraction through airlock mechanisms, can take as much as 1 h per sample. Therefore, screening tens to hundreds of individual NP fractions, a quantity that is routinely produced in an HPLC experiment on a crude NP extract, could require tens to hundreds of hours of TEM time and an equally large number of TEM grids. Recent advancements in the automated data collection for microED/3D ED have significantly increased the throughput of single-sample analysis, but the ability to routinely screen tens to hundreds of NP samples remains a challenge.^21−27^ This sample preparation bottleneck is shared among many methods that utilize TEMs, such as cryo-electron microscopy and tomography (cryoEM and cryoET).^28−33^ In this report, we describe a HT-screening workflow for microED inspired by Gianneschi and co-workers that enables the deposition of 96 or more unique samples onto a single TEM grid using noncontact printing of picoliter droplets from time-resolved HPLC fractions of NP extracts. (Figure 1c).^34,35^ This sample preparation workflow, which we coin ArrayED, enabled unambiguous structural characterization of 14 natural products from 20 crude extracts

## Results and Discussion

**Figure 2:**
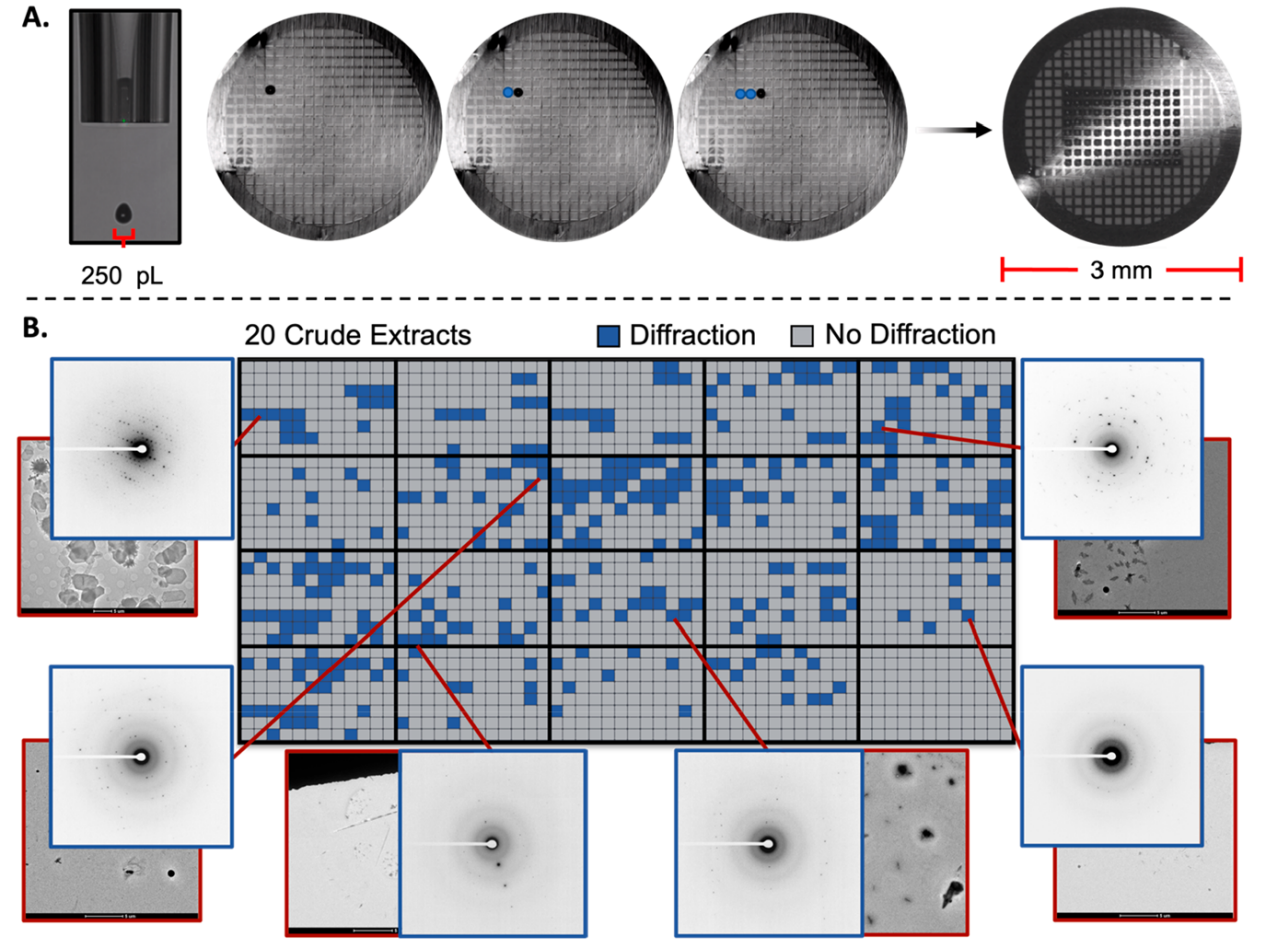
(A) Microarray printing and high-throughput screening of natural product extracts. Picoliter drops were utilized to print microarray and representation of microarrayed 96-well plate on a TEM grid. (B) Diffraction heatmap of 20 unique crude extracts within respective 96-well plates. Representative diffraction (blue dotted boxes) and associated microcrystals (red outlined boxes).

In the ArrayED workflow, crude extracts (obtained from plants, fungi, and marine organisms in this study) are divided onto a 96-well plate through generalized time-based HPLC fractionation. In initial experiments, we utilized a Phenomenex Kinetex 5 μM C18 column to employ a solvent gradient of 30% to 100% acetonitrile in H_2_O with 0.1% formic acid for 25 min at a flow rate of 4 mL per minute, thereby generating fractions every 15 s (96 fractions) that resulted in fractions of varying purities. This HPLC fractionating methodology was standardized on the basis of the ability to elute metabolites from multiple source organisms. Picoliter aliquots (250–350 pL) of each fraction were printed onto a single TEM grid (Figure 2a) in an automated and standardized fashion by utilizing a Scienion S3 microarrayer. To correlate the microdroplets deposited on the TEM grid to the positions from the 96-well plate, we incorporated a labeling system composed of saturated NaCl droplets deposited below the printed array to orient the top and bottom of the array (Figure S2). Each grid square corresponding to the individual well is visually inspected for particles using SerialEM, and snapshot diffraction is manually recorded for each identified particle.

In this proof-of-principle study, we were able to rapidly screen 20 extracts and their respective 1920 wells to classify fractions as diffracting or not with approximately 60 h of TEM time (Figure 2b). This process would have required 1000 h or more of TEM time, at least 1920 TEM grids (hundreds of thousands of dollars for TEM time and thousands of dollars in TEM grids) using standard microED workflows. Utilizing the ArrayED workflow, we identified 415 fractions containing crystalline materials (roughly 21% of all wells)—this classification includes monocrystalline, polycrystalline, low-resolution, and high-resolution diffraction. In cases where the observed diffraction was not suitable for direct methods solution (polycrystalline or low-resolution), subsequent crystallization was performed by dissolving isolated HPLC fractions in 60% acetonitrile in water and drop casting a 4 μL aliquot onto a new TEM grid for further study. For many fractions, improvements in diffraction quality could be obtained from larger droplets (4 μL vs 300 pL), presumably because of slower evaporation of solvent and enhanced dispersion of crystalline particles onto the grid. In general, the use of larger drop volumes resulted in higher quality data.

**Figure 3:**
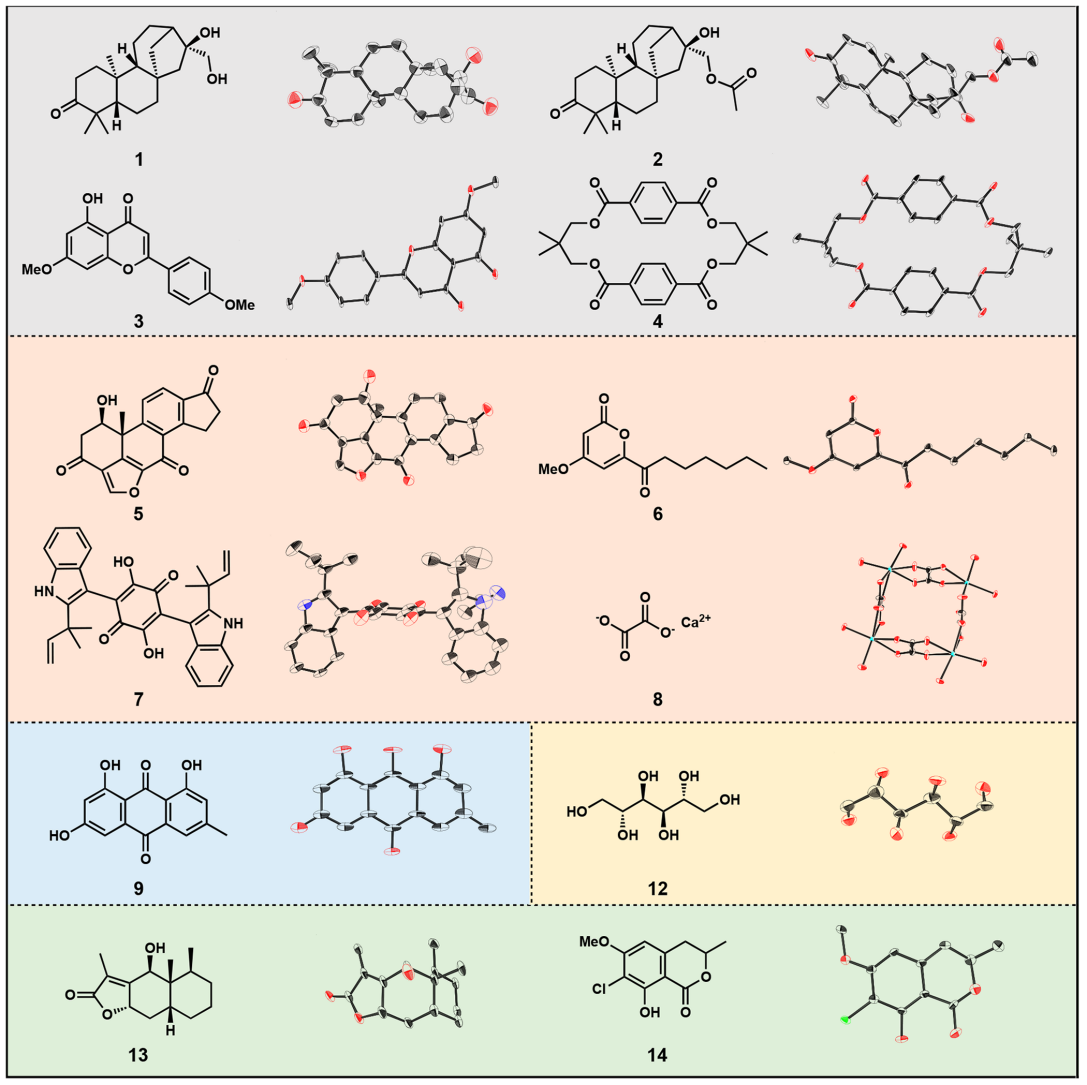
MicroED structures solved utilizing the microarraying diffraction technique. Thermal ellipsoids shaded with 30% probability and hydrogen atoms were omitted for clarity.

ArrayED analysis of an extract obtained from leaves of the American beautyberry (*Callicarpa americana*) resulted in the identification of 18 diffracting fractions of the total of 96 HPLC fractions collected. Ultimately, four structures were obtained from this extract. The crystal structure of calliterpenone (**1**) (Figure 3) was obtained directly from the microarrayed TEM grid.^36^ High-quality microcrystals of calliterpenone monoacetate (**2**) were obtained after additional recrystallization, which provided the first crystal structure of this complex diterpene natural product. The structure of a new polymorph of 5-hydroxy-4′,7-dimethoxy-flavone (**3**) was also resolved, as well as a polymorph of a presumed plasticizer phthalate **4**.^37^

As further validation of the method, a fungal extract obtained from *Hypoxylon himnuleum* was subjected to ArrayED, and 13 of the 96 HPLC fractions produced promising diffraction. With only 50 mg of crude extract, we ultimately solved the crystal structures of known fungal metabolites demethoxyviridin (**5**), xylariaopyrone A (**6**), hinnuliquinone (**7**), and calcium oxalate (**8**).^38−41^ For perspective, our workflow utilized a 500 mL liquid culture that is grown over 14–16 days and typically yields 50–150 mg of crude extract. Singh et al. reported their isolation of hinnuliquinone (**7**), a potent HIV-1 protease inhibitor, from ∼7.7 g of crude that required 28 days of cell culture. Moreover, isolation and purification of hinnuliquinone (**7**) required 15 iterative HPLC experiments to obtain sufficient material for structural characterization.^42^ In comparison with previous isolation efforts, ArrayED provided an unambiguous structure of hinnuliquinone from the crude extract in less time than it took to finish culturing the producing organism. Notably, all of the above metabolites (**5–8**) have no previously reported crystal structures, except for calcium oxalate (**8**).^43^

**Figure 4:**
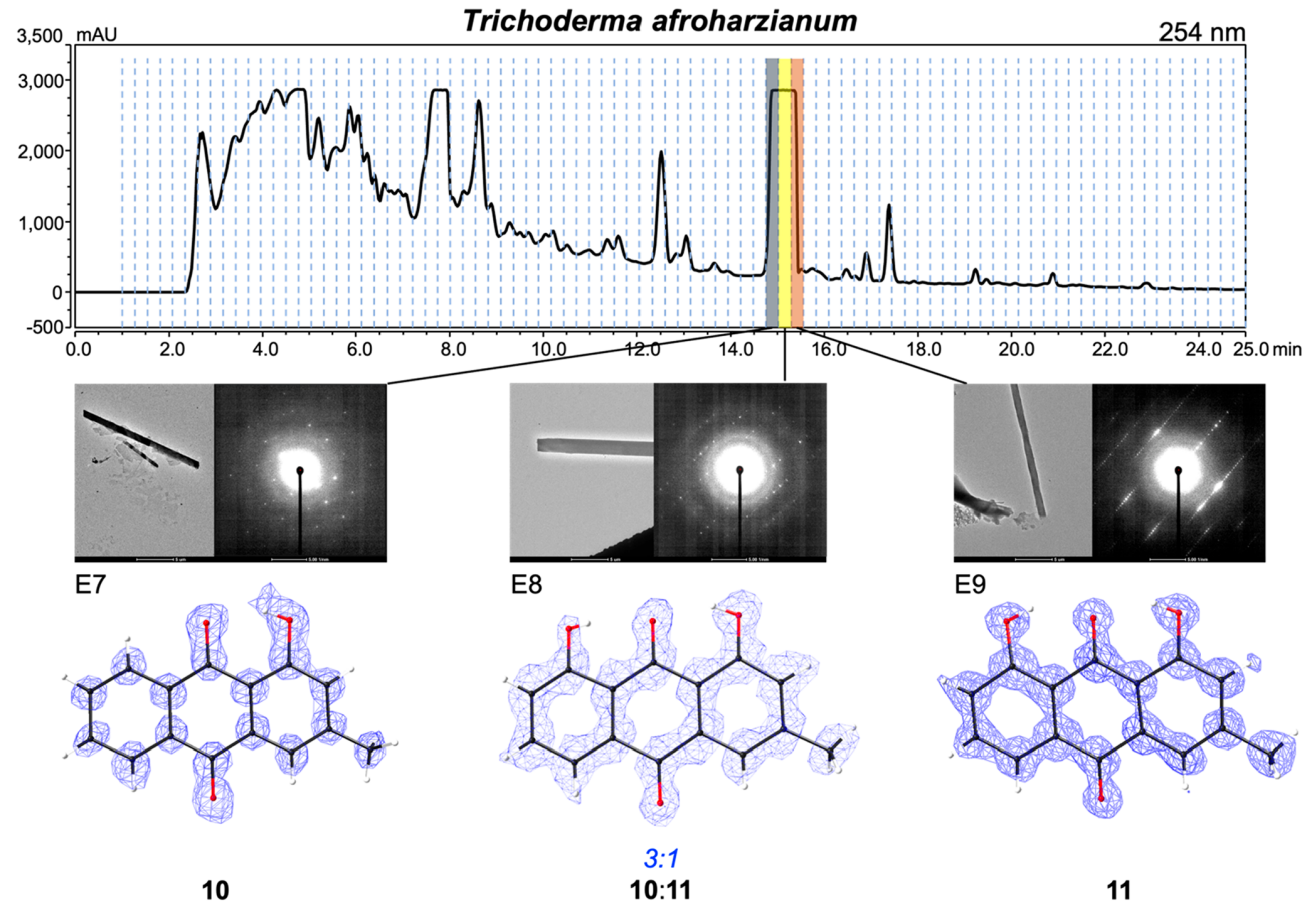
HPLC trace and images of adjacent wells containing pachybasin (**10**) and chrysophanol (**11**) and the three distinct polymorphs solved utilizing microED. The central well highlights the partially occupied oxygen (3:1) that distinguishes **10** from **11**.

We also studied a fungal extract of *Trichoderma afroharzianum*. Calcium oxalate (**8**) was also identified in this extract, in addition to three anthraquinone derivatives, emodin (**9**) (Figure 3), pachybasin (**10**) (Figure 4), and chrysophanol (**11**) (Figure 4). Interestingly, three unique crystal structures of pachybasin and chrysophanol were obtained from three adjacent wells from a single HPLC peak (Figure 4). Fractions E7 and E9 correspond to pure pachybasin and chrysophanol, respectively, while the middle fraction (E8) produced a structure containing mixed occupancy (3:1 of **10**/**11**) of the two species. This result supports our hypothesis that time-resolved fractionation conditions influence the crystallization of analytes on the basis of changes in concentration and the presence of impurities.

We further extended this method to study fungal extracts of *Preitonia* sp. and *Periconia* sp. Analysis of *Preitonia* sp. yielded the crystal structure of mannitol (**12**) (Figure 3), which reproduced a known polymorph.^44^ It is important to note that this metabolite may have not been identified if a peak-based approach was undertaken, as it is not active at standard UV wavelengths. Lastly, the analysis of *Periconia* sp. yielded the structures of 6β-hydroxyeremophilenolide (**13**) and 6-methoxy-7-chloromellein (**14**).

**Figure 5:**
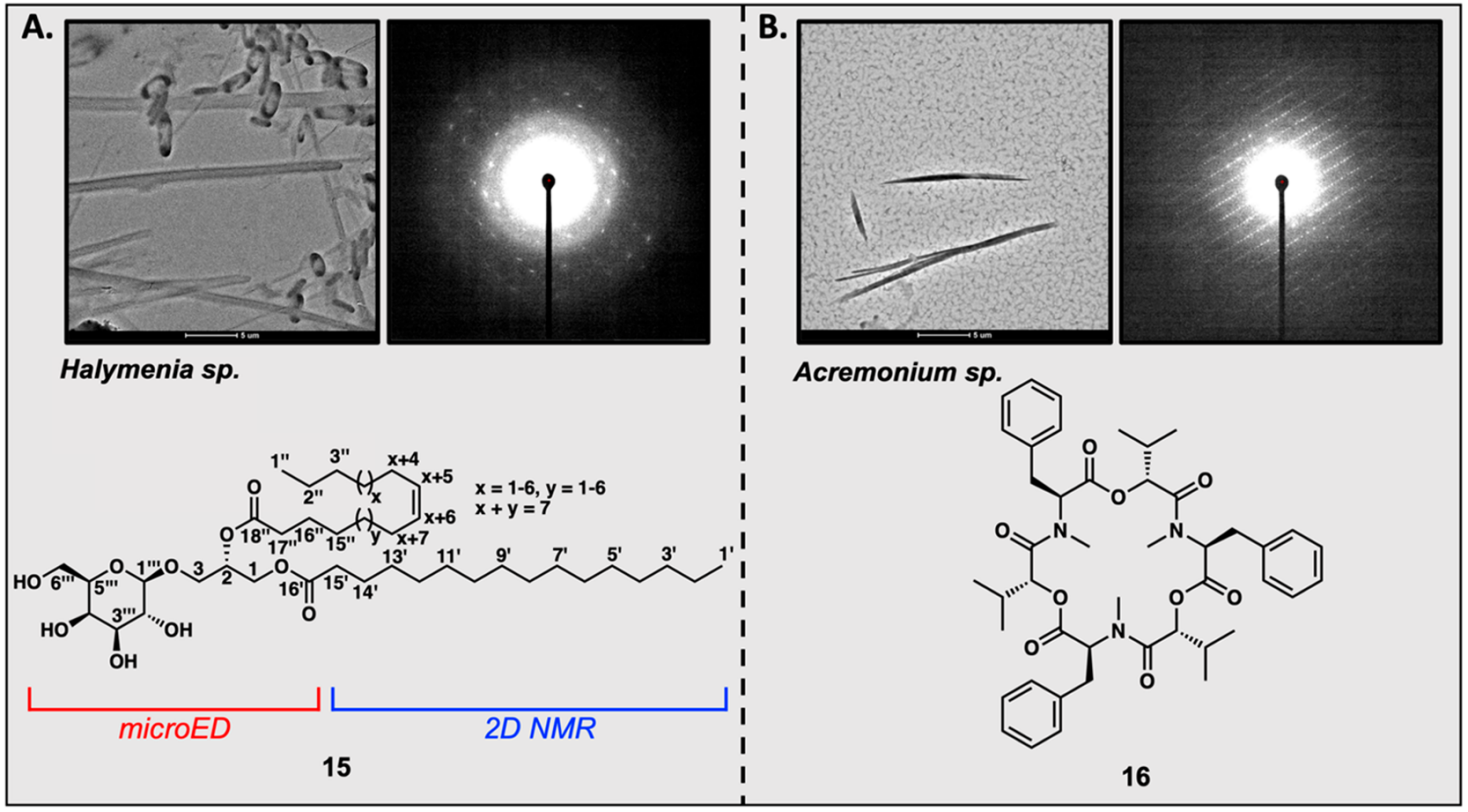
(A) Crystalline particles identified for **15** utilizing the ArrayED workflow and the structural identity of diffracting wells from *Halymenia* sp. utilizing microED, HRMS, and NMR for confirmation. (B) Crystalline particles identified for **16** and the structural identity of diffracting wells from *Acremonium* sp. utilizing microED, HRMS, and NMR for confirmation.

The ArrayED workflow can be utilized in conjunction with traditional NP characterization methods. While studying HPLC fractions of the marine red seaweed *Halymenia* sp. from Fiji, we identified several wells with crystalline particles. While many exhibited fibrous diffraction, well E11 contained a mixture of crystals with distinct morphologies, and each exhibited clean single-crystal diffraction (Figure 5A). MS and NMR spectroscopic analyses of isolated fractions suggested a glycosylated glycerolipid, which was initially proposed as a regioisomer of metabolite **15** (Figure 5A) with two fatty acyl chains exhibiting an *m*/*z* 774.6086 [M+NH_4_]^+^ consistent with the molecular formula C_43_H_80_O_10_. NMR spectra revealed chemical shifts and *J* coupling constants consistent with a β-d-galactose connected at C-3 of the glycerol. MS fragmentation confirmed a C-18 acyl chain (fragment ion *m*/*z* 339.2889) with one site of unsaturation and another saturated acyl chain with 16 carbons (palmitic acid, fragment ion *m*/*z* 313.2732). Because the carbon–carbon double bond was not within four carbons of its acyl headgroup, the ^1^H and ^13^C chemical shifts for the first four positions were identical for both fatty acyl chains, with the exception of the carbonyl ^13^C shifts. Three-bond heteronuclear multiple-bond correlation (HMBC) correlations from H_2_-1 and H-2 of the glycerol to their respective carbonyls were well resolved, but moving into the fatty acyl chains, identical methylene ^13^C and ^1^H signals precluded elucidation of the connectivity of each chain to the glycerol by either COSY or HMBC. A recrystallization of the diffracting well via slow evaporation afforded monocrystalline needles that yielded high-resolution microED data sets. A structural solution obtained via direct methods confirmed the 16- and 18-carbon fatty acyl chain lengths but identified the C-18 chain as attached to C-2 of the glycerol and the C-16 chain attached to C-1, thereby resolving the ambiguity of the NMR data. MicroED data also confirmed the relative and absolute configurations of the hexose sugar as galactose. Together, this combination of traditional (MS, NMR) and cutting-edge (microED) characterization methods allowed us to assign all bond connectivity. However, disorder within the crystal system prevented us from assigning a single site of unsaturation.

Similarly, we were able to identify well F9 from the fungal extract of *Acremonium* sp. as diffracting, but the crystalline species was extremely beam-sensitive, and a direct methods solution was not possible. Utilizing HRMS and 1D NMR data to make an initial structural assignment provided a plausible compound to compare our diffraction data against. Our comparison of unit cell dimensions obtained by our workflow did not match to that of published data on the CCDC. However, replication of the recrystallization procedures found in the literature generated a known polymorph and confirmed the natural product as beauvericin (**16**), a known metabolite previously isolated from *Beauveria bassiana* (Figure 5B).^45^ As exemplified by the rapid characterization of monogalactosyldiacylglyerides **15** and the identification of mycotoxin **16**, coupling ArrayED with traditional analytical methods can be a powerful approach to NP identification.^46−48^

## Conclusions

Here, we established the ArrayED workflow as a screening method to rapidly identify crystalline NPs from crude extracts. We leveraged this method, in addition to traditional analytical methods, to determine 14 structures from 20 extracts. In ArrayED, we utilized a mere 40–150 mg of crude extract to carry out the entire workflow in each example provided. We envision that adjacent biological screening of duplicate plates could rapidly associate the identified structures with a given bioactivity, thereby allowing ArrayED to become an integral part of the drug discovery process.

## Data Availability

The raw MicroED data of this study are available at 10.5281/zenodo.8206533, 10.5281/zenodo.10059575, 10.5281/zenodo.10059796, 10.5281/zenodo.10059842, 10.5281/zenodo.10059864, and 10.5281/zenodo.10064011. The auto processing python script used in this study is available at https://github.com/Jess-Burch/microED. CCDC 2246152–2246166 contains the supplementary crystallographic data for this paper. These data can be obtained free of charge via www.ccdc.cam.ac.uk/data_request/cif, or by emailing data_request@ccdc.cam.ac.uk, or by contacting The Cambridge Crystallographic Data Centre, 12 Union Road, Cambridge CB2 1EZ, UK; fax: + 44 1223 336033.

## Supporting Information Available

Detailed experimental protocols on sample preparation of fungal, plant, and marine algae extract into 96-well plate format; methods for microarraying 96-well plates onto 3 mm TEM grids; sample preparation and data collection methodology for microED; preliminary solutions and fully refined structural solutions for the identified natural products; Figures S1 and S2; and Tables S1 and S2 (PDF)

## Abbreviations

NP: natural product
HT: high-throughput
SCXRD: single-crystal X-ray diffraction
ED: electron diffraction
TEM: transmission electron microscopy
HPLC: high-performance liquid chromatography
MS: mass spectrometry
UV: ultraviolet
FTIR: Fourier transform infrared spectroscopy
NMR: nuclear magnetic resonance
3D ED: three-dimensional electron diffraction
MR: molecular replacement
ET: electron tomography
DNA: deoxyribonucleic acid

## References

[ref1] Newman D. J.; Cragg G. M. Natural products as sources of new drugs from 1981 to 2014. J. Nat. Prod. 2016, 79, 629–661. 10.1021/acs.jnatprod.5b01055. 26852623

[ref2] Newman D. J.; Cragg G. M. Natural products as sources of new drugs over the nearly four decades from 01/1981 to 09/2019. J. Nat. Prod. 2020, 83, 770–803. 10.1021/acs.jnatprod.9b01285. 32162523

[ref3] Boa A. N.; Jenkins P. R.; Lawrence N. J. Recent progress in the synthesis of taxanes. Contemp. Org. Syn. 1994, 1, 47–75. 10.1039/co9940100047.

[ref4] Dias D. A.; Urban S.; Roessner U. A historical overview of natural products in drug discovery. Metabolites 2012, 2, 303–336. 10.3390/metabo2020303. 24957513 PMC3901206

[ref5] Bucar F.; Wube A.; Schmid M. Natural product isolation - how to get from biological material to pure compounds. Nat. Prod. Rep. 2013, 30, 525–545. 10.1039/c3np20106f. 23396532

[ref6] Wilson B. A. P.; Thornburg C. C.; Henrich C. J.; Grkovic T.; O’Keefe B. R. Creating and screening natural product libraries. Nat. Prod. Rep. 2020, 37, 893–918. 10.1039/C9NP00068B. 32186299 PMC8494140

[ref7] Hanson J. R. Natural products: the secondary metabolites, Royal Society of Chemistry: Cambridge, United Kingdom, 2003.

[ref8] Yoo H. D.; Nam S. J.; Chin Y. W.; Kim M. S. Arch. of Pharmacal Res. 2016, 39, 143–153. 10.1007/s12272-015-0649-9. 26310208

[ref9] Chhetri B. K.; Lavoie S.; Sweeney-Jones A. M.; Kubanek J. Recent trends in the structural revision of natural products. Nat. Prod. Rep. 2018, 35, 514–531. 10.1039/C8NP00011E. 29623331 PMC6013367

[ref10] Reisberg S. H.; Gao Y.; Walker A. S.; Helfrich E. J. N.; Clardy J.; Baran P. S. Total synthesis reveals atypical atropisomerism in a small-molecule natural product, tryptorubin A. Science 2020, 367, 458–463. 10.1126/science.aay9981. 31896661 PMC6996792

[ref11] Vainshtein B. K.; Feigl E.; Spink J. A. Structure analysis by electron diffraction; Elsevier Science: Burlington, MA, 2013.

[ref12] Kim L. J.; Ohashi M.; Zhang Z.; Tan D.; Asay M.; Cascio D.; Rodriguez J. A.; Tang Y.; Nelson H. M. Prospecting for natural product structural complexity using genome mining and microcrystal electron diffraction. Nat. Chem. Biol. 2021, 17, 872–877. 10.1038/s41589-021-00834-2. 34312563 PMC8447837

[ref13] Jones C. G.; Martynowycz M. W.; Hattne J.; Fulton T. J.; Stoltz B. M.; Rodriguez J. A.; Nelson H. M.; Gonen T. The cryoEM method MicroED as a powerful tool for small molecule structure determination. ACS Cent. Sci. 2018, 4, 1587–1592. 10.1021/acscentsci.8b00760. 30555912 PMC6276044

[ref14] Gruene T.; Wenmacher J. T. C.; Zaubitzer C.; Holstein J. J.; Heidler J.; Fecteu-Lefebvre A.; De Carlo S.; Müller E.; Goldie K. N.; Regeni I.; Li T.; Santiso-Quinones G.; Steinfeld G.; Handschin S.; Van Genderen E.; van Bokhoven J. A.; Clever G. H.; Pantelic R.; et al. Rapid structure determination of microcrystalline molecular compounds using electron diffraction. Angew. Chem., Int. Ed. 2018, 57, 16313–16317. 10.1002/anie.201811318. PMC6468266 30325568

[ref15] Gemmi M.; Mugnaioli E.; Gorelik T. E.; Kolb U.; Palatinus L.; Boullay P.; Hovmoller S.; Abrahams J. P. 3D electron diffraction: the nanocrystallography revolution. ACS Cent. Sci. 2019, 5, 1315–1329. 10.1021/acscentsci.9b00394. 31482114 PMC6716134

[ref16] Danelius E.; Halaby S.; van der Donk W. A.; Gonen T. MicroED in natural product and small molecule research. Nat. Prod. Rep. 2021, 38, 423–431. 10.1039/D0NP00035C. 32939523 PMC7965795

[ref17] Saha A.; Nia S. S.; Rodriguez J. A. Electron diffraction of 3D molecular crystals. Chem. Rev. 2022, 122, 13883–13914. 10.1021/acs.chemrev.1c00879. 35970513 PMC9479085

[ref18] Kim L. J.; Xue M.; Li X.; Xu Z.; Paulson E.; Mercado B.; Nelson H. M.; Herzon S. B. Structure revision of the lomaiviticins. J. Am. Chem. Soc. 2021, 143, 6578–6585. 10.1021/jacs.1c01729. 33900077 PMC8935351

[ref19] Luft J. R.; Wolfley J.; Jurisica I.; Glasgow J.; Fortier S.; DeTitta G. T. Macromolecular crystallization in a high throughput laboratory-the search phase. J. Cryst. Growth 2001, 232, 591–595. 10.1016/S0022-0248(01)01206-4.

[ref20] Barnes C. O.; Kovaleva E. G.; Fu X.; Stevenson H. P.; Brewster A. S.; DePonte D. P.; Baxter E. L.; Cohen A. E.; Calero G. Assessment of microcrystal quality by transmission electron microscopy for efficient serial femtosecond crystallography. Arch. Biochem. Biophys. 2016, 602, 61–68. 10.1016/j.abb.2016.02.011. 26944553 PMC5215478

[ref21] Smeets S.; Zou X.; Wan W. Serial electron crystallography for structure determination and phase analysis of nanocrystalline materials. J. Appl. Crystallogr. 2018, 51, 1262–1273. 10.1107/S1600576718009500. 30279637 PMC6157704

[ref22] Cichocka M. O.; Angstrom J.; Wang B; Zou X.; Smeets S. High-throughput continuous rotation electron diffraction data acquisition via software automation. J. Appl. Crystallogr. 2018, 51, 1652–1661. 10.1107/S1600576718015145. 30546290 PMC6276279

[ref23] de la Cruz M. J.; Martynowycz M. W.; Hattne J.; Gonen T. MicroED data collection with SerialEM. Ultramicroscopy. 2019, 201, 77–80. 10.1016/j.ultramic.2019.03.009. 30986656 PMC6752703

[ref24] Wang B.; Zou X.; Smeets S. Automated serial rotation electron diffraction combined with cluster analysis: an efficient multi-crystal workflow for structure determination. IUCrJ. 2019, 6, 854–867. 10.1107/S2052252519007681. 31576219 PMC6760450

[ref25] Bucker R.; Hogan-Lamarre P.; Mehrabi P.; Schulz E. C.; Bultema L. A.; Gevorkov Y.; Brehm W.; Yefanov O.; Oberthur D.; Kassier G. H.; Miller R. J. D. Serial protein crystallography in an electron microscope. Nat. Commun. 2020, 11, 996 10.1038/s41467-020-14793-0. 32081905 PMC7035385

[ref26] Takaba K.; Maki-Yonekura S.; Yonekura K. Collecting large datasets of rotational electron diffraction with ParallEM and SerialEM. J. Struct. Biol. 2020, 211, 107549 10.1016/j.jsb.2020.107549. 32544623

[ref27] Luo Y.; Wang B.; Smeets S.; Sun J.; Yang W.; Zou X. High-throughput phase elucidation of polycrystalline materials using serial rotation electron diffraction. Nat. Chem. 2023, 15, 483–490. 10.1038/s41557-022-01131-8. 36717616 PMC10070184

[ref28] Danev R.; Yanagisawa H.; Kikkawa M. Cryo-electron microscopy methodology: current aspects and future directions. Trends Biochem. Sci. 2019, 44, 837–848. 10.1016/j.tibs.2019.04.008. 31078399

[ref29] Wu M.; Lander G. C. Present and emerging methodologies in cryo-EM single-particle analysis. Biophys. J. 2020, 119, 1281–1289. 10.1016/j.bpj.2020.08.027. 32919493 PMC7567993

[ref30] Bohning J.; Bharat T. A. M. Towards high-throughput in situ structural biology using electron cryotomography. Prog. Biophys. Mol. Biol. 2021, 160, 97–103. 10.1016/j.pbiomolbio.2020.05.010. 32579969

[ref31] Mulligan S. K.; Speir J. A.; Razinkov I.; Cheng A.; Crum J.; Jain T.; Duggan E.; Liu E.; Nolan J. P.; Carragher B.; Potter C. S. Multiplexed TEM specimen preparation and analysis of plasmonic nanoparticles. Microsc. Microanal. 2015, 21, 1017–1025. 10.1017/S1431927615014324. 26223550 PMC4701052

[ref32] Arnold S. A.; Albiez S.; Opara N.; Chami M.; Schmidli C.; Bieri A.; Padeste C.; Stahlberg H.; Braun T. Total sample conditioning and preparation of nanoliter volumes for electron microscopy. ACS Nano 2016, 10, 4981–4988. 10.1021/acsnano.6b01328. 27074622

[ref33] Castro-Hartmann P.; Heck G.; Eltit J. M.; Fawcett P.; Samso M. The ArrayGrid: a methodology for applying multiple samples to a single TEM specimen grid. Ultramicroscopy 2013, 135, 105–112. 10.1016/j.ultramic.2013.07.014. 23954856

[ref34] Touve M. A.; Wright D. B.; Mu C.; Sun H.; Park C.; Gianneschi N. C. Block Copolymer Amphiphile Phase Diagrams by High-Throughput Transmission Electron Microscopy. Macromolecules 2019, 52, 5529–5537. 10.1021/acs.macromol.9b00563.

[ref35] Gong X.; Gnanasekaran K.; Ma K.; Forman C. J.; Wang X.; Su S.; Farha O.; Gianneschi N. C. Rapid Generation of Metal-Organic Framework Phase Diagrams by High-Throughput Transmission Electron Microscopy. J. Am. Chem. Soc. 2022, 144, 6674–6680. 10.1021/jacs.2c01095. 35385280

[ref36] Wong W. H.; Wei C.; Loke S. E.; Mak T. C. W. Structure of calliterpenone hemihydrate. Acta Crystallogr. 1991, C47, 906–908. 10.1107/S0108270190010332.

[ref37] Davila A.; McLaughlin M. L.; Fronczek F. R. CCDC 957988. CSD Communication 2013, 10.5517/cc114vvw.

[ref38] Aldridge D. C.; Turner B. W.; Geddes A. J.; Sheldrick B. Demethoxyviridin and demethoxyviridiol: new fungal metabolites. J. Chem. Soc., Perkin Trans. 1 1975, 10, 943–945. 10.1039/p19750000943.

[ref39] Guo Z. Y.; Lu L. W.; Bao S. S.; Liu C. X.; Deng Z. S.; Cao F.; Liu S. P.; Zou K.; Proksch P. Xylariaopyrones A-D, four new antimicrobial α-pyrone derivatives from endophytic fungus *Xylariales sp*. Phytochem. Lett. 2018, 28, 98–103. 10.1016/j.phytol.2018.09.014.

[ref40] O’Leary M. A.; Hanson J. R.; Yeoh B. L. The structure and biosynthesis of hinnuliquinone, a pigment from *Nodulisporium hinnuleum*. J. Chem. Soc., Perkin Trans. 1984, 1, 567–569. 10.1039/p19840000567.

[ref41] Graustein W. C.; Cromack K. Jr.; Sollins P. Calcium oxalate: occurrence in soils and effect on nutrient and geochemical cycles. Science 1977, 198, 1252–1254. 10.1126/science.198.4323.1252. 17741705

[ref42] Singh S. B.; Ondeyka J. G.; Tsipouras N.; Ruby C.; Sardana V.; Schulman M.; Sanchez M.; Pelaez F.; Stahlhut M. W.; Munshi S.; Olsen D. B.; Lingham R. B. Hinnuliquinone, a C_2_-symmetric dimeric non-peptide fungal metabolite inhibitor of HIV-1 protease. Biochem. Biophys. Res. Commun. 2004, 324, 108–113. 10.1016/j.bbrc.2004.08.234. 15464989

[ref43] Sterling C. Crystal structure analysis of weddellite, CaC_2_O_4_•(2+x)H_2_O. Acta Crystallogr. 1965, 18, 917 10.1107/S0365110X65002219.

[ref44] Kim H. S.; Jeffrey G. A.; Rosenstein R. D. The crystal structure of the K form of D-mannitol. Acta Crystallogr. 1968, B24, 1449 10.1107/S0567740868004462.

[ref45] Mallebrera B.; Prosperini A.; Font G.; Ruiz M. J. In vitro mechanisms of Beauvericin toxicity: A review. Food Chem. Toxicol. 2018, 111, 537–545. 10.1016/j.fct.2017.11.019. 29154952

[ref46] Mizushina Y.; Sugiyama Y.; Yoshida H.; Hanashima S.; Yamazaki T.; Kamisuki S.; Ohta K.; Takemura M.; Yamaguchi T.; Matsukage A.; Yoshida S.; Saneyoshi M.; Sugawara F.; Sakagauchi K. Galactosyldiacylglycerol, a mammalian DNA polymerase alpha-specific inhibitor from a sea alga, Petalonia bingbamiae. Biol. Pharm. Bull. 2001, 24, 982–987. 10.1248/bpb.24.982. 11558581

[ref47] Hamill R. L.; Higgens C. E.; Boaz H. E.; Gorman M. The structure of beauvericin, a new depsipeptide antibiotic toxic to Artemia salina. Tetrahedron Lett. 1969, 10, 4255–4258. 10.1016/S0040-4039(01)88668-8.

[ref48] Hyuk-Hwan S.; Ahn J.; Lim Y. H.; Lee C. Analysis of beauvericin and unusual enniatins co-produced by *Fusarium oxysporum* FB1501(KFCC 11363P). J. Microbiol. Biotechnol. 2006, 16, 1111–1119.

