# Supplementary material for: High-Throughput Identification of Crystalline Natural Products from Crude Extracts Enabled by Microarray Technology and microED
Source: ACS Cent Sci. 2023 Dec 20;10(1):176–83. doi: 10.1021/acscentsci.3c01365 (PMC10823509; doi:10.1021/acscentsci.3c01365)
Supplement: Supplementary file 1 — oc3c01365_si_001.pdf [file oc3c01365_si_001.pdf]

Supporting Information for

High-throughput identification of crystalline natural products from crude extracts enabled by microarray technology and microED

David A. Delgadillo[†], Jessica E. Burch[†], Lee Joon Kim[‡], Lygia S. de Moraes[†], Kanji Niwa[§], Jason Williams[‡], Melody J. Tang[†], Vincent G. Lavallo[†], Bhuwan Khatri Chhetri[||], Christopher G. Jones[†], Isabel Hernandez Rodriguez[†], Joshua A. Signore[†], Lewis Marquez[⊥], Riya Bhanushali[||], Sunmin Woo[¶], Julia Kubanek[||*], Cassandra Quave[⊥¶#*], Yi Tang[‡§*], Hosea M. Nelson[†*]

[†]Division of Chemistry and Chemical Engineering, California Institute of Technology, Pasadena, California 91125, United States.

[‡]Department of Chemistry and Biochemistry, [§]Department of Chemical and Biomolecular Engineering, University of California, Los Angeles, Los Angeles, California 90095, United States.

[||]School of Biological Sciences, School of Chemistry and Biochemistry, and Neuroscience Program, Georgia Institute of Technology, Atlanta, Georgia 30332, United States.

[⊥]Molecular and Systems Pharmacology, Laney Graduate School, Emory University, Atlanta, Georgia 30322, United States.

[¶]Center for the Study of Human Health, Emory University, Atlanta, Georgia 30322, United States.

[#]Department of Dermatology, Emory University School of Medicine, Atlanta, Georgia 30322, United States.

[*]Correspondence to:

Email: hosea@caltech.edu (H.M.N.)

Email: yitang@g.ucla.edu (Y.T.)

Email: julia.kubanek@biosci.gatech.edu (J.K.)

Email: cassandra.leah.quave@emory.edu (C.Q.)

Supporting Information

Table of Contents

1. Materials and Methods……………………………………………………………………S3

1.1. Fungal Extracts to 96-Well Plate Protocol

1.2 Plant Extract to 96-Well Plate Protocol

1.3 Marine Algae Extract to 96-Well Plate Protocol

2. Microarraying of 96-Well Plates to 3 mm TEM Grids ………………………………......S7

3. Sample Preparation………………...S8

4. Microcrystal Electron Diffraction Natural Product Structures ……….....................……S9

5. References…………………..S56

1. Materials and Methods

1.1 Fungal Extracts to 96-Well Plate Protocol:

Strain Culturing

The strains utilized in this study were maintained on PDA (potato dextrose agar, BD) for 3 days for sporulation or in liquid PDB medium (PDA medium without agar) for isolation of genomic DNA. Plugs of mycelium were then placed directly into broth media (MMK2, PDB, or PDB SS) for inoculation. The liquid cultures were placed on a shaker and allowed to grow for 14 days.

Extraction and Isolation

The liquid culture was then filtered using cheesecloth and the resulting filtrate was extracted with an equal volume of ethyl acetate (2x wash). The combined extract was filtered and concentrated in vacuo by rotary evaporation and weighed. The extract was then dissolved in methanol to a concentration between 100-200 mg/L and subjected to HPLC separation using a mobile phase isocratic flow of 30% acetonitrile in water for 1 minute followed by a gradient to 100% acetonitrile in water over 20 minutes (solvent flow rate = 4 ml/min). 96 fractions were generated utilizing 15 second time slices and deposited into deep-well plates. The solvents were allowed to dry via slow evaporation and promptly sealed for shipment.

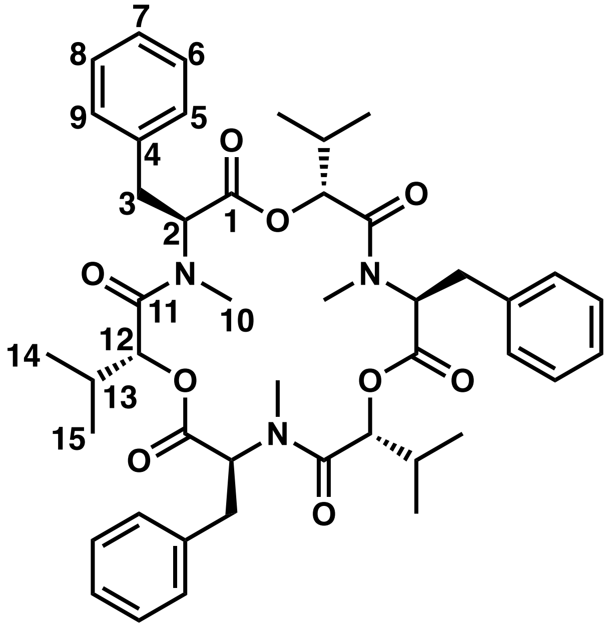

Table S1. NMR spectral data for beauvericin **16** (Bruker 600 MHz, $CDCl_3$).

Position #	δ_C	$\delta_{H\ (mult,\ J\ Hz)}$
1	169.4	–
2	57.3	5.57 (1H, brs)
3	34.8	3.38, 2.99 (each 1H, m)

4	136.7	–
5	128.9	7.27 (1H, m)
6	128.6	7.27 (1H, m)
7	126.8	7.20 (1H, t, 5.8)
8	128.6	7.27 (1H, m)
9	128.9	7.27 (1H, m)
10	170.0	
11	75.5	4.94 (1H, d, 8.5)
12	29.7	2.03 (1H, m)
13	18.3	0.45 (3H, d, 6.5)
14	17.5	0.82 (3H, d, 6.5)
N-Me	32.4	3.02 (3H, s)

1.2. Plant Extract to 96-Well Plate Protocol

Specimen Collection and Identification

The air-dried leaves of *C. americana* L. were collected in Atlanta, GA, USA in June and August 2017, and from The Jones Center at Ichuaway in Baker County, GA, USA in June 2018 and 2019. The identification of the plant was done by botanist Dr. Tharanga Samarakoon, Emory University Herbarium. A voucher specimen (accession numbers 22044, 22205, 22848, and 25009) is deposited in the Emory University Herbarium (GEO). Vouchers are available for viewing through the SERNEC portal.[1] Leaves were dried in a dehumidified chamber and ground to powder in a Wiley Mill (Thomas Scientific, Swedesboro, NJ) with a 2 mm mesh.

Extraction and isolation

Air-dried leaves of *C. americana* L. (522.0 g), powdered by Wiley Mill (Thomas Scientific, Swedesboro, NJ) with a 2 mm mesh, was double macerated in 95% ethanol at a 1:10 ratio (w/v) with daily agitation at room temperature for 72 h for each time. The combined extract was filtered and concentrated in vacuo by rotary evaporation and lyophilization. Dried extract was stored at -20 °C.[2]

1.3. Marine Algae Extract to 96-Well Plate Protocol

General Experimental Procedures

Preparative TLC separation was performed with Silicycle (20 × 20 cm, 200 μm thickness) thin-layer chromatography (TLC) plates. HPLC separations were carried out using a Waters 1525 binary pump connected to a normal phase silica (250×4.6 mm; 5 μm particle size) column. Altech

ELSD 800 (for method development) and Waters 2996 Photodiode Array detectors were used to monitor HPLC separations. NMR spectral data (1D and 2D) were acquired with a 18.8 T (800 MHz for ^{1}H and 201 MHz for ^{13}C) Bruker Avance IIIHD instrument equipped with a ultra-high sensitivity 3 mm TCI helium temperature cryoprobe. All NMR data were recorded in DMSO-d_6. HRMS data were acquired with a Thermo Scientific IDX Tribrid mass spectrometer.

Specimen Collection and Identification

The red alga *Halymenia* sp. (G-0815) was collected as part of an NIH ICBG natural products discovery program off Viti Levu Island, Fiji (S 18°14.184′, E 177°46.86′) in April 2010. The red alga was found growing on reef slope and overhangs at depths of 5–40 m. *Halymenia* sp. had a pink-red color and soft texture with low mucus. After comparing its morphological traits with the literature, the organism was determined to be of the genus *Halymenia* but of uncertain species.[3] The bulk sample of the alga, which was used for extraction, was frozen and stored at −80°C at Georgia Institute of Technology, where formalin vouchers are also stored.

Extraction and isolation

Halymenia sp. (428.2 g wet weight) was exhaustively extracted with methanol, 1:1 methanol/DCM, and DCM, with extracts combined and dried in vacuo to get yield 17.4 g of crude extract. A 1:9 mixture of water/methanol was used to suspend the crude extract and partitioned with hexanes. The 1:9 water/methanol portion was adjusted to 2:3 water/methanol (by adding water) and partitioned with DCM. The water/methanol portion was evaporated *in vacuo* to remove methanol and the remaining water-soluble materials were partitioned with ethyl acetate. Hence, liquid/liquid partition resulted in four fractions: F1 (hexanes-soluble), F2 (DCM-soluble), F3 (ethyl acetate-soluble), and F4 (water-soluble) fractions. A portion of fraction F2 (0.7 g) was subjected to silica gel prep TLC (in multiple batches) chromatographed using a 1:4 methanol/ethyl acetate mobile phase system resulting in 8 fractions (with F2.1 being the most polar). A portion of fraction F2.7 (15.4 mg) was subjected to normal phase silica (250×4.6 mm; 5 μm particle size) HPLC separation using a mobile phase gradient of ethyl acetate to 60% MeOH over 20 min (solvent flow rate = 1 ml/min). Forty fractions were collected at 30 second intervals. While fractions 1–11, 18–32, 33–34, 35–36, 37–38, and 39–40 were combined based on ^{1}H NMR data, fractions 12, 13, 14, 15, 16, and 17 were kept separate. Fractions were dissolved in 2 ml MeOH and 100 μl was transferred into a 96-well clear round bottom cell culture microplate, dried, and submitted for Micro ED. Based on ^{1}H NMR spectroscopic data, fractions 14 and 15 contained the same compound. While fraction 14 generated crystals suitable for Micro ED, fraction 15 was used for 1D, 2D NMR, and HRMS based characterization.

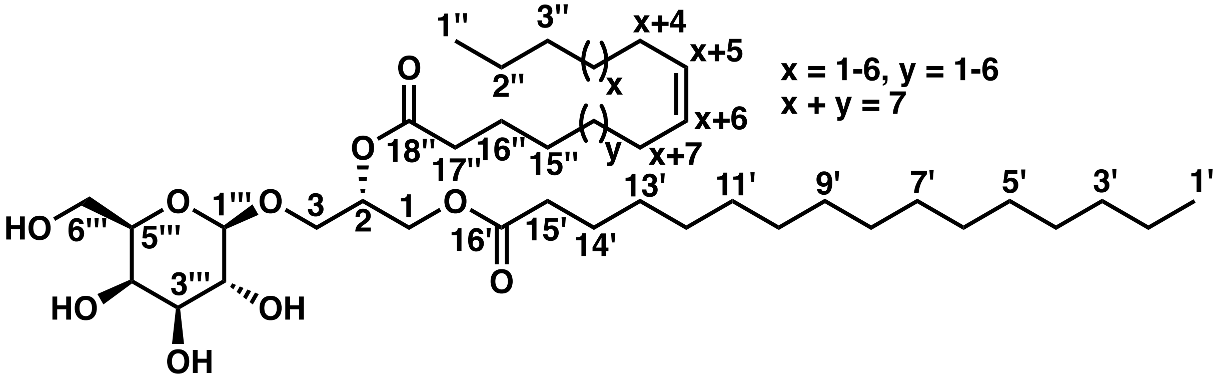

Table S2. NMR spectral data for glycosylated glycerolipid **15** (800 MHz, DMSO-*d6*).

Position #	δ_C	δ_H (mult, *J* Hz)	COSY	HMBC
1a	62.4 (CH_2)	4.14 dd (12.0, 7.1)	H-2	C-2, C-3, C-16′
1b		4.32 dd (12.0, 2.9)	H-2	C-3, C-16′
2	70.0 (CH)	5.10 m	H-1, H-3a, H-3b	C-1, C-3, C-18″
3a	66.7 (CH_2)	3.60 m	H-2	C-1, C-2, C-1‴
3b		3.82 dd (10.9, 5.5)	H-2	C-1, C-2, C-1‴
1′	13.9 (CH_3)	0.85 dd (6.9, 6.9)	H-2′	C-2′, C-3′
1″	13.9 (CH_3)	0.85 dd (6.9, 6.9)	H-2″	C-2″, C-3″
2a′	22.1 (CH_2)	1.28 m	H-1′	C-1′
2b′		1.28 m	H-1′	C-1′
2a″	22.1 (CH_2)	1.28 m	H-1″	C-1″
2b″		1.28 m	H-1″	C-1″
3a′	31.3 (CH_2)	1.23 m		
3b′		1.23 m		
3a″	31.3 (CH_2)	1.23 m		
3b″		1.23 m		
4a′–13a′	28.4–29.1 (CH_2)	1.23 br s	14a′, 14b′	C-14′
4b′–13b′		1.23 br s	14a′, 14b′	C-14′
x, y, 15a″	28.4–29.1 (CH_2)	1.23 br s	H-(x+4)a, H-(x+4)b, H-(x+7)a, H-(x+7)b, 16a″, 16b″	C-16″
x, y, 15b″	28.4–29.1 (CH_2)	1.23 br s	H-(x+4)a, H-(x+4)b, H-(x+7)a, H-(x+7)b, 16a″, 16b″	C-16″
14a′	24.4 (CH_2)	1.49 m	H-13a′, H-13b′, H-15a′, H-15b′	C-13′, C-15′, C-16′
14b′		1.49 m	H-13a′, H-13b′, H-15a′, H-15b′	C-13′, C-15′, C-16′
15a′	33.4/33.6 (CH_2)	2.24–2.30 m	H-14a′, H-14b′	C-13′, C-14′, C-16′
15b′		2.24–2.30 m	H-14a′, H-14b′	C-13′, C-14′, C-16′
16a″	24.4 (CH_2)	1.49 m	H-15a″, H-15b″, H-17a″, H-17b″	C-15″, C-17″, C-18″
16b″		1.49 m	H-15a″, H-15b″, H-17a″, H-17b″	C-15″, C-17″, C-18″
17a″	33.4/33.6 (CH_2)	2.24–2.30 m	H-16a″, H-16b″	C-15″, C-16″, C-18″
17b″		2.24–2.30 m	H-16a″, H-16b″	C-15″, C-16″, C-18″
x+6	129.5/129.6	5.31 pseudo t (5.0)	H-(x+7)a, H-(x+7)b	C-(x+7)
x+5	129.5/129.6	5.31 pseudo t (5.0)	H-(x+4)a, H-(x+4)b	C-(x+4)
(x+7)a	26.6	1.97 m	H-(x+6), H-(y)	C-(x+6), C-(y)
(x+7)b		1.97 m	H-(x+6), H-(y)	C-(x+6), C-(y)
(x+4)a	26.6	1.97 m	H-(x+5), H-(x)	C-(x+5), C-(x)
(x+4)b		1.97 m	H-(x+5), H-(x)	C-(x+5), C-(x)
16′	172.6 (C)			
18″	172.3 (C)			
1‴	103.8 (CH)	4.09 d (7.3)	H-2‴	C-3, C-3‴
2‴	70.4 (CH)	3.26 m	H-1‴, H-3‴, OH (4.85)	C-1‴C-3‴
3‴	73.3 (CH)	3.24 m	H-2‴, H-4‴, OH (4.70)	C-2‴
4‴	68.0 (CH)	3.62 m	H-3‴, H-5‴, OH (4.36)	C-3‴, C-2‴

5‴	75.3 (CH)	3.32 m	H-6a‴, H-6b‴	C-1‴, C-4‴, C-6‴
6a‴	60.3 (CH_2)	3.46 m	H-5‴, OH (4.53)	C-4‴, C-5‴
6b‴		3.52 m	H-5‴, OH (4.53)	C-4‴, C-5‴
	OH	4.36 d (4.6)	H-4‴	C-4‴, C-5‴
	OH	4.53 dd (5.6, 5.6)	H-6a‴, H-6b‴	C-5‴, C-6‴
	OH	4.70 d (5.2)	H-3‴	C-2‴, C-3‴, C-4‴
	OH	4.85 d (4.5)	H-2‴	C-2‴, C-3‴

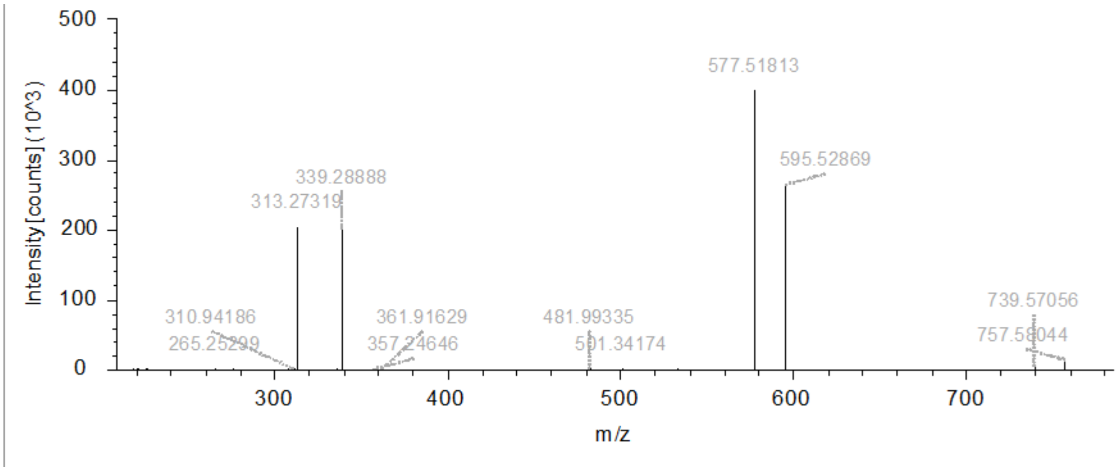

Figure S1. MS2 data for the glycosylated glycerolipid **15**.

2. Microarraying of 96-Well Plates to 3 mm TEM Grids using Scienion S3

sciFLEXARRAYER S3 is a piezoelectric liquid handling robot that enables automated non- contact dispensing of solutions with volumes in the picoliter to microliter range. The 350 µL of each fractionated well samples contained in a 96-well polypropylene DeepWell plate were transferred to a shallow 96-well polypropylene or glass-coated plate. The sciFLEXARRAYER S3 system was equipped with a PDC 60 Type 3 capable of depositing drop volume between 250–350 pL. The microarrayer was operated at 30% humidity, and the deck and probe plate temperatures were controlled to be at the dew point (~11 °C). Prior to the runs, nozzle and camera were aligned, and the grids were placed on a glass slide resting next to a spacer on the deck. The grids were manually aligned using the head camera to ensure that the grid squares were perpendicular to the X and Y axes of the microarrayer. After the alignment, two runs were utilized for each full 96-well plates: the first run deposits the 96 samples onto a grid, followed by a second run to label the array with saturated NaCl (**Figure S2**).

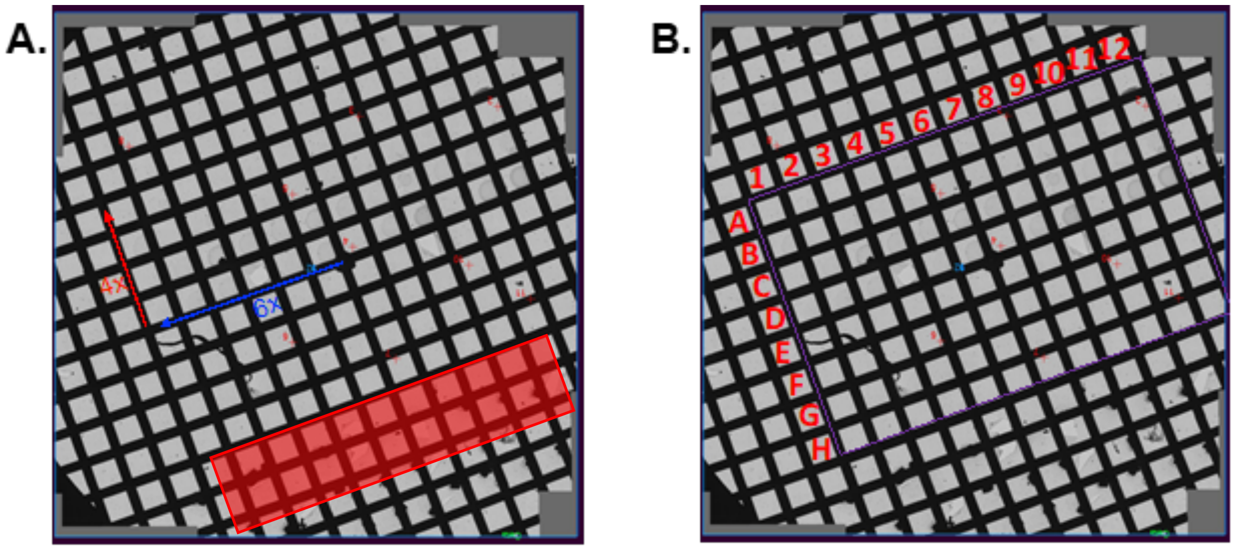

Figure S2. (A) Micrograph of a 96 well microarray with the red rectangle depicting the NaCl region that informs the orientation of our array. From the NaCl region on can then navigate to the center of the grid, followed by a move of six grid squares west of the center and four grid squares north. (B) Representation of the 96 well microarray post orientation.

3. Sample Preparation

Samples prepared according to previously disclosed procedures outlined in Jones et. al, utilizing 3.0mm copper grids with 200 mesh and Formvar/carbon support film.[4] Data was collected on a Thermo Fisher Talos Arctica transmission electron microscope equipped with an Autoloader and operating with an accelerating voltage of 200keV at 80 Kelvin, corresponding to an electron wavelength of 0.0251 Å. Electron diffraction data was collected using a Thermo Fisher CetaD camera with a 960 mm detector distance. Screening the TEM grid for microcrystals was performed at 2600x magnification in imaging mode. Particles were visually selected for data collection and isolated by a selected area aperture. Data was collected by taking images of the diffraction patterns generated by a continuously rotating crystal integrated continuously at a rate of 3 seconds per frame. This rotation was performed at a rate of 0.3° per second with a minimum and maximum tilt range of −70° to +70°. Crystals selected for data collection were isolated by a selected area aperture of 100 μm to reduce the background noise contributions and calibrated to eucentric height to stay in the aperture over the entire tilt range. All diffraction data was processed using the XDS suite of programs as controlled by a custom Python automation script.[5–7] Structures were solved *ab initio* by direct methods in SHELXT or SHELXD and the direct preliminary solution is included for each entry. After this, structures were refined with SHELXL using ShelXle and incorporating electron scattering factors.[8–11] Thermal parameters were refined anisotropically for all non-hydrogen atoms. Hydrogen atoms were assigned using the riding model unless otherwise noted.

4. Microcrystal Electron Diffraction Natural Product Structures

4.1 hexane-1,2,3,4,5,6-hexaol (SI-1, CCDC 2246165).

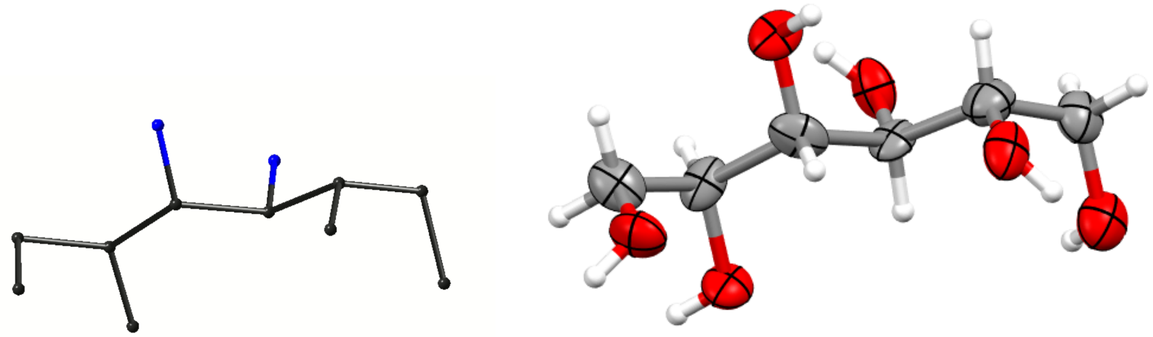

Initial direct methods solution of **SI-1** (left) and ORTEP diagram of refined **SI-1** (right). Thermal ellipsoids shown as shaded octants at 30% probability.

Crystal data and structure refinement for SI-1.

Empirical formula	$C_6H_{14}O_6$
Formula weight	182.17

Data Collection

Type of instrument	Talos Arctica F200C
Wavelength	0.0215 Å
Data collection temperature	80(4) K
Unit cell dimensions	a = 4.9500(10)
	b = 9.060(2)
	c = 19.270(4)
Volume	864.2(3)
Z	4
Crystal system	Orthorhombic

Space group	$P2_12_12_1$
Density (calculated)	1.400 Mg/m^3
F(000)	12
Measured reflections	921
Reflections with $I > 2\sigma(I)$	485
Resolution	0.95 Å
Completeness	82.9%
Index ranges	5 ≤ h ≤ -5, 9 ≤ k ≤ -9, 18 ≤ l ≤ -18

Structure Solution and Refinement

Structure solution program	SHELXT (Uson & Sheldrick, 1999)
Primary solution method	Direct methods
Secondary solution method	Difference Fourier map
Hydrogen placement	Geometric positions
Structure refinement program	SHELXL-2018/3 (Sheldrick, 2018)
Refinement method	Full matrix least-squares on F^2
Data / restraints / parameters	921 / 66 / 116
Treatment of hydrogen atoms	Riding
Goodness-of-fit on F^2	1.045
Final R indices [I>2s(I)]	R1 = 0.1266, *w*R2 = 0.3286
R indices (all data)	R1 = 0.1769, *w*R2 = 0.3733
Type of weighting scheme used	Sigma

Weighting scheme used	$w=1/s^2(Fo^2)$
Max shift/error	0.000
Average shift/error	0.000
Largest diff. peak and hole	0.08 and -0.10 e.Å^{-3}

Special Refinement Details

Refinement of F^2 against ALL reflections. The weighted R-factor (wR) and goodness of fit (S) are based on F-^{2}, conventional R-factors (R) are based on F, with F set to zero for negative F^2. The threshold expression of $F^2 > 2s(F^2)$ is used only for calculating R-factors(gt) etc. and is not relevant to the choice of reflections for refinement. R-factors based on F^2 are statistically about twice as large as those based on F, and R-factors based on ALL data will be even larger.

All esds (except the esd in the dihedral angle between two l.s. planes) are estimated using the full covariance matrix. The cell esds are taken into account individually in the estimation of esds in distances, angles and torsion angles; correlations between esds in cell parameters are only used when they are defined by crystal symmetry. An approximate (isotropic) treatment of cell esds is used for estimating esds involving l.s. planes.

4.2 calcium oxalate dihydrate (SI-2, CCDC 2246152).

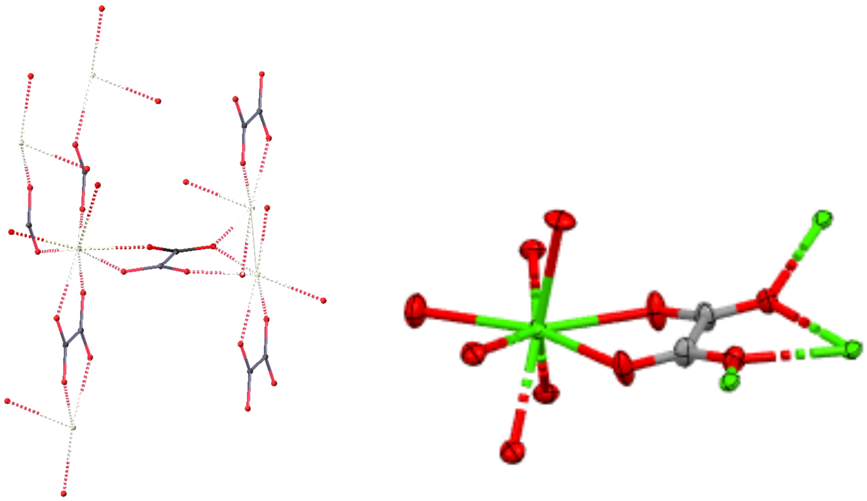

Expanded initial direct methods solution of **SI-2** (left) and expanded ORTEP diagram of refined **SI-2** (right). Thermal ellipsoids shown as shaded octants at 30% probability.

Crystal data and structure refinement for SI-2.

Empirical formula	C_2O_6Ca
Formula weight	160.10

Data Collection

Type of instrument	Talos Arctica F200C
Wavelength	0.0215 Å
Data collection temperature	80(4) K
Unit cell dimensions	a = 12.3600(10)
	b = 12.360(2)
	c = 7.400(4)
Volume	1130.5(7)
Z	8
Crystal system	Tetragonal

Space group	I4/m
Density (calculated)	1.881 Mg/m^3
F(000)	19
Measured reflections	392
Reflections with I > 2σ(*I*)	224
Resolution	0.90 Å
Completeness	87.7%
Index ranges	13 ≤ h ≤ -13, 13 ≤ k ≤ -13, 7 ≤ l ≤ -7

Structure Solution and Refinement

Structure solution program	SHELXT (Uson & Sheldrick, 1999)
Primary solution method	Direct methods
Secondary solution method	Difference Fourier map
Hydrogen placement	Difference Fourier map
Structure refinement program	SHELXL-2018/3 (Sheldrick, 2018)
Refinement method	Full matrix least-squares on F^2
Data / restraints / parameters	392 / 12 / 47
Goodness-of-fit on F^2	1.142
Final R indices [I>2s(I)]	R1 = 0.1285, *w*R2 = 0.3113
R indices (all data)	R1 = 0.1768, *w*R2 = 0.3448
Type of weighting scheme used	Sigma
Weighting scheme used	$w=1/s^2(Fo^2)$

Max shift/error	0.000
Average shift/error	0.000
Largest diff. peak and hole	0.25 and -0.25 e.$Å^{-3}$

Special Refinement Details

Refinement of F^2 against ALL reflections. The weighted R-factor (wR) and goodness of fit (S) are based on F^{-2}, conventional R-factors (R) are based on F, with F set to zero for negative F^2. The threshold expression of $F^2 > 2s(F^2)$ is used only for calculating R-factors(gt) etc. and is not relevant to the choice of reflections for refinement. R-factors based on F^2 are statistically about twice as large as those based on F, and R-factors based on ALL data will be even larger.

All esds (except the esd in the dihedral angle between two l.s. planes) are estimated using the full covariance matrix. The cell esds are taken into account individually in the estimation of esds in distances, angles and torsion angles; correlations between esds in cell parameters are only used when they are defined by crystal symmetry. An approximate (isotropic) treatment of cell esds is used for estimating esds involving l.s. planes.

4.3 5-hydroxy-4',7-dimethoxy-flavone (**SI-3, CCDC 2246162).**

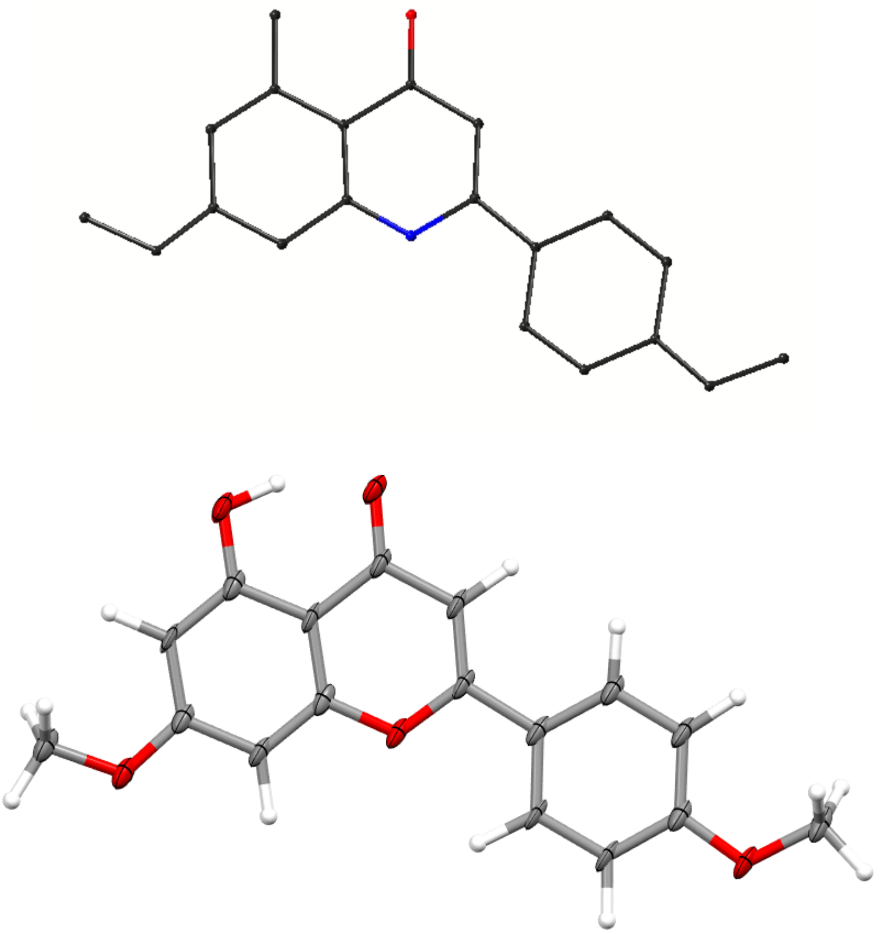

Initial direct methods solution of **SI-3** (top) and ORTEP diagram of refined **SI-3** (bottom). Thermal ellipsoids shown as shaded octants at 30% probability.

Crystal data and structure refinement for SI-3.

Empirical formula	$C_{17}H_{15}O_5$
Formula weight	298.28

Data Collection

Type of instrument	Talos Arctica F200C
Wavelength	0.0215 Å

Data collection temperature	80(4) K
Unit cell dimensions	a = 17.1100(10)
	b = 3.830(2)
	c = 21.910(4)
	β□= 106.79□
Volume	1374.5(8)
Z	4
Crystal system	Monoclinic
Space group	$P2_1/c$
Density (calculated)	1.441 Mg/m^3
F(000)	10
Measured reflections	2100
Reflections with I > 2σ(*I*)	1278
Resolution	0.85 Å
Completeness	86.7%
Index ranges	20 ≤ h ≤ -20, 4 ≤ k ≤ -4, 24 ≤ l ≤ -24

Structure Solution and Refinement

Structure solution program	SHELXT (Uson & Sheldrick, 1999)
Primary solution method	Direct methods
Secondary solution method	Difference Fourier map
Structure refinement program	SHELXL-2018/3 (Sheldrick, 2018)

Refinement method	Full matrix least-squares on F^2
Data / restraints / parameters	2100 / 388 / 243
Treatment of hydrogen atoms	refxyz
Goodness-of-fit on F^2	1.421
Final R indices [I>2s(I)]	R1 = 0.1523, *w*R2 = 0.3952
R indices (all data)	R1 = 0.1977, *w*R2 = 0.4167
Type of weighting scheme used	Sigma
Weighting scheme used	$w=1/s^2(Fo^2)$
Max shift/error	0.005
Average shift/error	0.000
Largest diff. peak and hole	0.21 and -0.17 e.Å^{-3}

Special Refinement Details

Refinement of F^2 against ALL reflections. The weighted R-factor (wR) and goodness of fit (S) are based on $F-^2$, conventional R-factors (R) are based on F, with F set to zero for negative F^2. The threshold expression of $F^2 > 2s(F^2)$ is used only for calculating R-factors(gt) etc. and is not relevant to the choice of reflections for refinement. R-factors based on F^2 are statistically about twice as large as those based on F, and R-factors based on ALL data will be even larger.

All esds (except the esd in the dihedral angle between two l.s. planes) are estimated using the full covariance matrix. The cell esds are taken into account individually in the estimation of esds in distances, angles and torsion angles; correlations between esds in cell parameters are only used when they are defined by crystal symmetry. An approximate (isotropic) treatment of cell esds is used for estimating esds involving l.s. planes.

4.4 7-chloro-8-hydroxy-6-methoxy-3-methylisochroman-1-one (SI-4, CCDC 2246166).

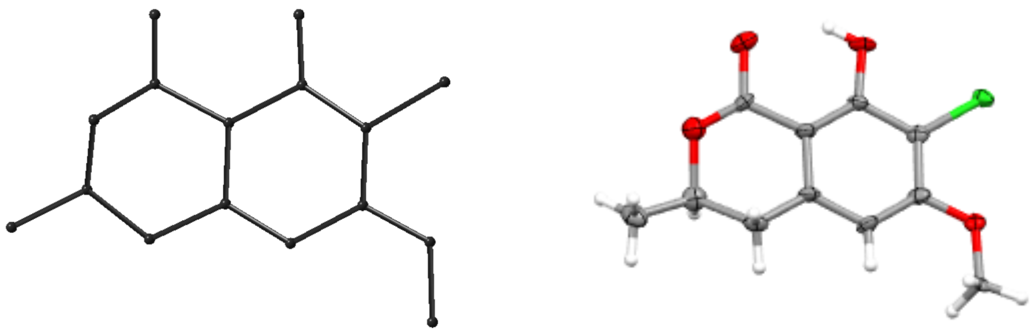

Initial direct methods solution of **SI-4** (left) and ORTEP diagram of refined **SI-4** (right). Thermal ellipsoids shown as shaded octants at 30% probability.

Crystal data and structure refinement for SI-4.

Empirical formula	$C_{11}H_{11}ClO_4$
Formula weight	242.65

Data Collection

Type of instrument	Talos Arctica F200C
Wavelength	0.0215 Å
Data collection temperature	80(4) K
Unit cell dimensions	a = 4.9300(10)
	b = 5.850(2)
	c = 18.780(4)
	β = 96.02
Volume	538.6(2)
Z	2

Crystal system	Monoclinic
Space group	$P2_1$
Density (calculated)	1.496 Mg/m^3
F(000)	0
Measured reflections	1827
Reflections with $I > 2\sigma(I)$	1072
Resolution	0.85 Å
Completeness	83.3%
Index ranges	$6 \leq h \leq -6$, $7 \leq k \leq -7$, $20 \leq l \leq -20$

Structure Solution and Refinement

Structure solution program	SHELXD (Uson & Sheldrick, 1999)
Primary solution method	Direct methods
Secondary solution method	Difference Fourier map
Structure refinement program	SHELXL-2018/3 (Sheldrick, 2018)
Refinement method	Full matrix least-squares on F^2
Data / restraints / parameters	1827 / 229 / 147
Treatment of hydrogen atoms	Riding
Goodness-of-fit on F^2	1.274
Final R indices [I>2s(I)]	R1 = 0.1499, *w*R2 = 0.3896
R indices (all data)	R1 = 0.1999, *w*R2 = 0.4232
Type of weighting scheme used	Sigma

Weighting scheme used	$w=1/s^2(Fo^2)$
Max shift/error	0.056
Average shift/error	0.000
Largest diff. peak and hole	0.18 and -0.22 e.Å^{-3}

Special Refinement Details

Refinement of F^2 against ALL reflections. The weighted R-factor (wR) and goodness of fit (S) are based on F-^{2}, conventional R-factors (R) are based on F, with F set to zero for negative F^2. The threshold expression of $F^2 > 2s(F^2)$ is used only for calculating R-factors(gt) etc. and is not relevant to the choice of reflections for refinement. R-factors based on F^2 are statistically about twice as large as those based on F, and R-factors based on ALL data will be even larger.

All esds (except the esd in the dihedral angle between two l.s. planes) are estimated using the full covariance matrix. The cell esds are taken into account individually in the estimation of esds in distances, angles and torsion angles; correlations between esds in cell parameters are only used when they are defined by crystal symmetry. An approximate (isotropic) treatment of cell esds is used for estimating esds involving l.s. planes.

4.5 7- 4-hydroxy-3,4a,5-trimethyl-4a,5,6,7,8,8a,9,9a-octahydronaphtho[2,3-b]furan-2(4H)-one (SI-5, CCDC 2246154).

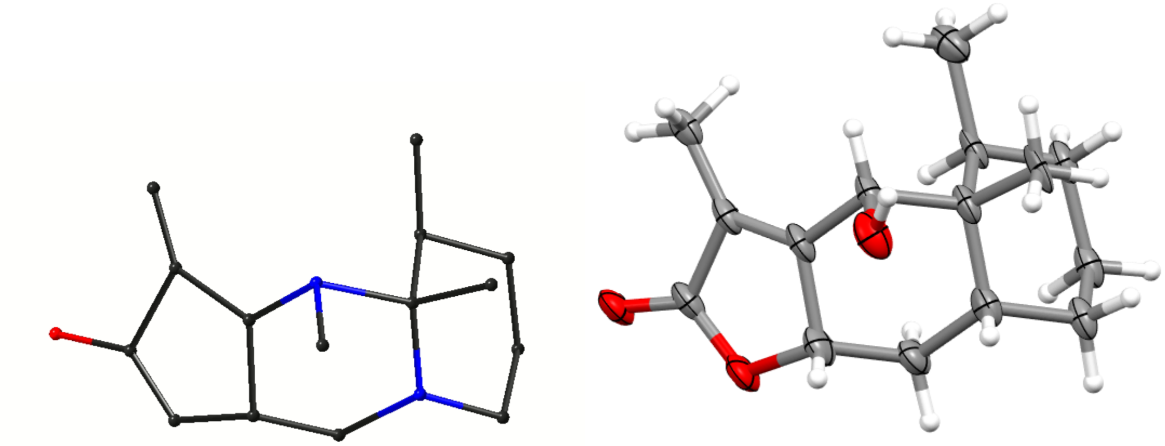

Initial direct methods solution of **SI-5** (left) and expanded ORTEP diagram of refined **SI-5** (right). Thermal ellipsoids shown as shaded octants at 30% probability.

Crystal data and structure refinement for SI-5.

Empirical formula	$C_{15}H_{22}O_3$
Formula weight	250.33

Data Collection

Type of instrument	Talos Arctica F200C
Wavelength	0.0215 Å
Data collection temperature	80(4) K
Unit cell dimensions	a = 7.1200(10)
	b = 13.360(2)
	c = 28.500(4)
Volume	2711.0(7)
Z	8

Crystal system	Orthorhombic
Space group	$C222_1$
Density (calculated)	1.227 Mg/m^3
F(000)	5
Measured reflections	1745
Reflections with $I > 2\sigma(I)$	1112
Resolution	0.90 Å
Completeness	88.5%
Index ranges	$7 \leq h \leq -7$, $13 \leq k \leq -13$, $31 \leq l \leq -31$

Structure Solution and Refinement

Structure solution program	SHELXT (Uson & Sheldrick, 1999)
Primary solution method	Direct methods
Secondary solution method	Difference Fourier map
Structure refinement program	SHELXL-2018/3 (Sheldrick, 2018)
Refinement method	Full matrix least-squares on F^2
Data / restraints / parameters	1745 / 328 / 165
Treatment of hydrogen atoms	Riding
Goodness-of-fit on F^2	1.393
Final R indices [I>2s(I)]	R1 = 0.1534, *w*R2 = 0.3782
R indices (all data)	R1 = 0.1933, *w*R2 = 0.4034
Type of weighting scheme used	Sigma

Weighting scheme used	$w=1/s^2(Fo^2)$
Max shift/error	0.077
Average shift/error	0.002
Largest diff. peak and hole	0.17 and -0.20 e.Å^{-3}

Special Refinement Details

Refinement of F^2 against ALL reflections. The weighted R-factor (wR) and goodness of fit (S) are based on F-^{2}, conventional R-factors (R) are based on F, with F set to zero for negative F^2. The threshold expression of $F^2 > 2s(F^2)$ is used only for calculating R-factors(gt) etc. and is not relevant to the choice of reflections for refinement. R-factors based on F^2 are statistically about twice as large as those based on F, and R-factors based on ALL data will be even larger.

All esds (except the esd in the dihedral angle between two l.s. planes) are estimated using the full covariance matrix. The cell esds are taken into account individually in the estimation of esds in distances, angles and torsion angles; correlations between esds in cell parameters are only used when they are defined by crystal symmetry. An approximate (isotropic) treatment of cell esds is used for estimating esds involving l.s. planes.

4.6 1-hydroxy-11b-methyl-1,7,8,11b-tetrahydrocyclopenta[7,8]phenanthro[10,1-bc]furan-3,6,9(2H)-trione (SI-6, CCDC 2246158).

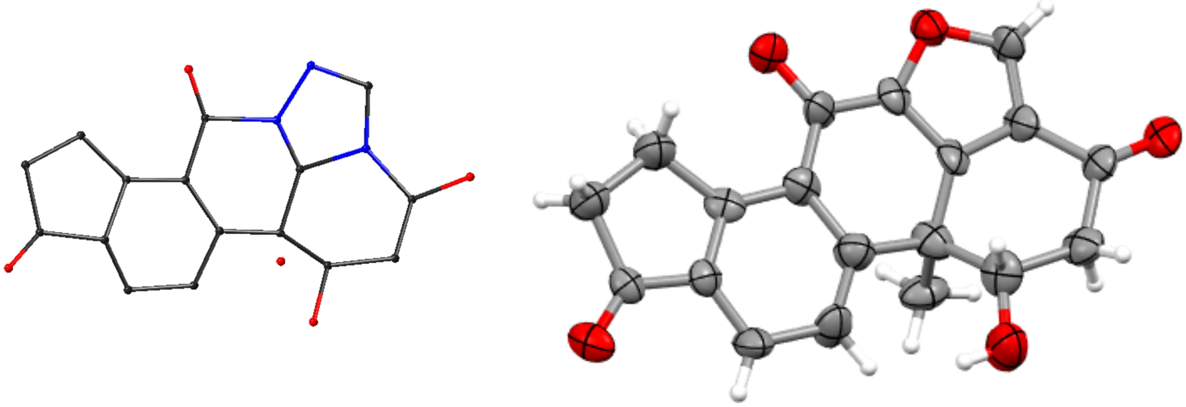

Initial direct methods solution of **SI-6** (left) and ORTEP diagram of refined **SI-6** (right). Thermal ellipsoids shown as shaded octants at 30% probability.

Crystal data and structure refinement for SI-6.

Empirical formula	$C_{19}H_{14}O_5$
Formula weight	322.30

Data Collection

Type of instrument	Talos Arctica F200C
Wavelength	0.0215 Å
Data collection temperature	80(4) K
Unit cell dimensions	a = 21.300(4)
	b = 6.5700(10)
	c = 10.820(2)
Volume	1514.2(5)
Z	4

Crystal system	Orthorhombic
Space group	$P2_12_12$
Density (calculated)	1.227 Mg/m^3
F(000)	0
Measured reflections	1394
Reflections with $I > 2\sigma(I)$	825
Resolution	1.0 Å
Completeness	86.2%
Index ranges	21 ≤ h ≤ -21, 6 ≤ k ≤ -6, 10 ≤ l ≤ -10

Structure Solution and Refinement

Structure solution program	SHELXT (Uson & Sheldrick, 1999)
Primary solution method	Direct methods
Secondary solution method	Difference Fourier map
Structure refinement program	SHELXL-2018/3 (Sheldrick, 2018)
Refinement method	Full matrix least-squares on F^2
Data / restraints / parameters	1394 / 242 / 219
Treatment of hydrogen atoms	Riding
Goodness-of-fit on F^2	1.178
Final R indices [I>2s(I)]	R1 = 0.1575, *w*R2 = 0.3816
R indices (all data)	R1 = 0.2020, *w*R2 = 0.4227
Type of weighting scheme used	Sigma

Weighting scheme used	$w=1/s^2(Fo^2)$
Max shift/error	0.024
Average shift/error	0.000
Largest diff. peak and hole	0.13 and -0.13 e.Å^{-3}

Special Refinement Details

Refinement of F^2 against ALL reflections. The weighted R-factor (wR) and goodness of fit (S) are based on F-^{2}, conventional R-factors (R) are based on F, with F set to zero for negative F^2. The threshold expression of $F^2 > 2s(F^2)$ is used only for calculating R-factors(gt) etc. and is not relevant to the choice of reflections for refinement. R-factors based on F^2 are statistically about twice as large as those based on F, and R-factors based on ALL data will be even larger.

All esds (except the esd in the dihedral angle between two l.s. planes) are estimated using the full covariance matrix. The cell esds are taken into account individually in the estimation of esds in distances, angles and torsion angles; correlations between esds in cell parameters are only used when they are defined by crystal symmetry. An approximate (isotropic) treatment of cell esds is used for estimating esds involving l.s. planes.

4.7 2,5-dihydroxy-3,6-bis(2-(2-methylbut-3-en-2-yl)-1H-indol-3-yl)cyclohexa-2,5-diene-1,4-dione (SI-7, CCDC 2246156).

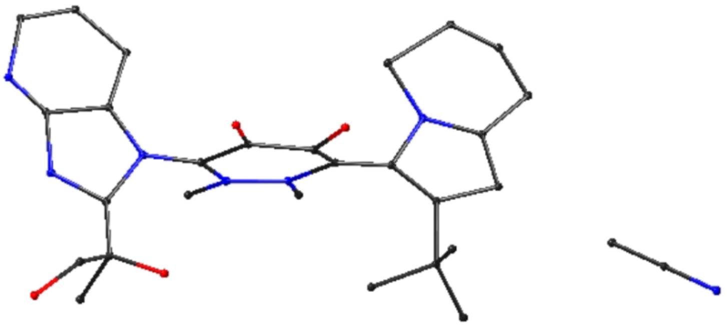

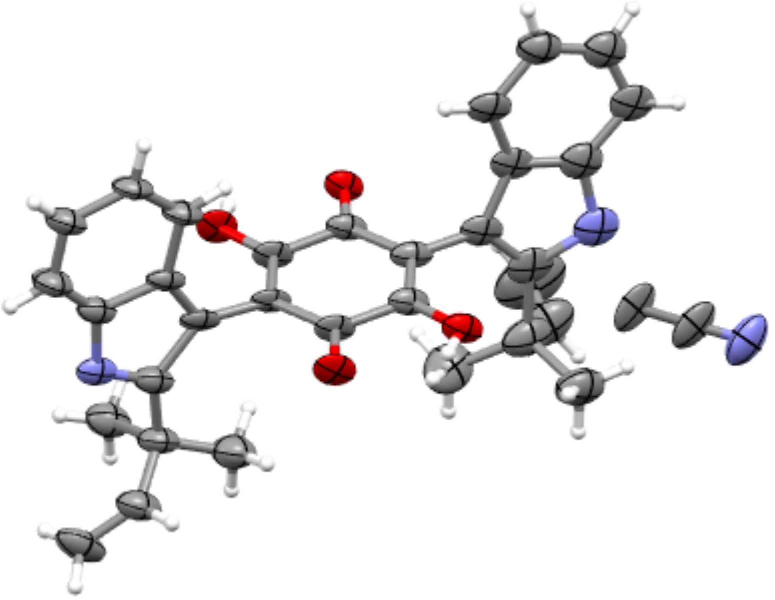

Initial direct methods solution of **SI-7** (top) and ORTEP diagram of refined **SI-7** (bottom). Thermal ellipsoids shown as shaded octants at 30% probability.

Crystal data and structure refinement for SI-7.

Empirical formula	$C_{34}H_{28}N_3O_4$
Formula weight	542.59

Data Collection

Type of instrument	Talos Arctica F200C
Wavelength	0.0215 Å
Data collection temperature	80(4) K
Unit cell dimensions	a = 7.3500(10)
	b = 8.650(2)
	c = 24.560(4)
	α□= 89.97□
	β□= 96.27□
	γ□= 106.32
Volume	1488.8(5)
Z	2
Crystal system	Triclinic
Space group	P-1
Density (calculated)	1.210 Mg/m^3
F(000)	12
Measured reflections	2705
Reflections with I > 2σ(*I*)	1460
Resolution	1.0 Å
Completeness	86.5%
Index ranges	7 ≤ h ≤ -7, 8 ≤ k ≤
	-8, 23 ≤ l ≤ -23

Structure Solution and Refinement

Structure solution program	SHELXD (Uson & Sheldrick, 1999)
Primary solution method	Direct methods
Secondary solution method	Difference Fourier map
Structure refinement program	SHELXL-2018/3 (Sheldrick, 2018)
Refinement method	Full matrix least-squares on F^2
Data / restraints / parameters	2705 / 598 / 373
Treatment of hydrogen atoms	Riding
Goodness-of-fit on F^2	1.643
Final R indices [I>2s(I)]	R1 = 0.2305, *w*R2 = 0.5112
R indices (all data)	R1 = 0.2896, *w*R2 = 0.5410
Type of weighting scheme used	Sigma
Weighting scheme used	$w=1/s^2(Fo^2)$
Max shift/error	0.002
Average shift/error	0.000
Largest diff. peak and hole	0.26 and -0.18 e.Å^{-3}

Special Refinement Details

Refinement of F^2 against ALL reflections. The weighted R-factor (wR) and goodness of fit (S) are based on F-^{2}, conventional R-factors (R) are based on F, with F set to zero for negative F^2. The threshold expression of $F^2 > 2s(F^2)$ is used only for calculating R-factors(gt) etc. and is not relevant to the choice of reflections for refinement. R-factors based on F^2 are statistically about twice as large as those based on F, and R-factors based on ALL data will be even larger.

All esds (except the esd in the dihedral angle between two l.s. planes) are estimated using the full covariance matrix. The cell esds are taken into account individually in the estimation of esds in distances, angles and torsion angles; correlations between esds in cell parameters are only used when they are defined by crystal symmetry. An approximate (isotropic) treatment of cell esds is used for estimating esds involving l.s. planes.

4.8 8-hydroxy-4,4,11b-trimethyl-3-oxotetradecahydro-6a,9-methanocyclohepta[a]naphthalen-8-yl)methyl acetate (SI-8, CCDC 2246153).

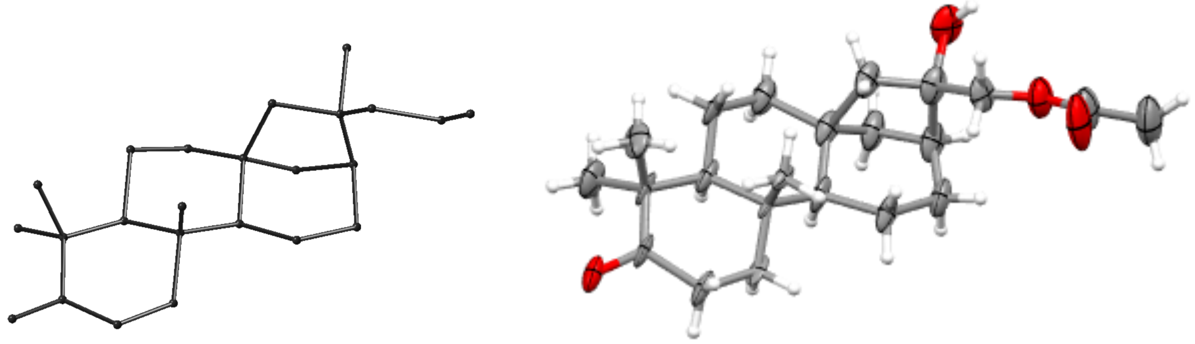

Initial direct methods solution of **SI-8** (left) and ORTEP diagram of refined **SI-8** (right). Thermal ellipsoids shown as shaded octants at 30% probability.

Crystal data and structure refinement for SI-8.

Empirical formula	$C_{22}H_{34}O_4$
Formula weight	362.49

Data Collection

Type of instrument	Talos Arctica F200C
Wavelength	0.0215 Å
Data collection temperature	80(4) K
Unit cell dimensions	a = 13.8800(10)
	b = 6.320(2)
	c = 23.950(4)
	β = 106.10
Volume	2018.5(7)
Z	4

Crystal system	Monoclinic
Space group	C2
Density (calculated)	1.193 Mg/m^3
F(000)	10
Measured reflections	1740
Reflections with I > 2σ(*I*)	1131
Resolution	1.0 Å
Completeness	81.8%
Index ranges	13 ≤ h ≤ -13, 6 ≤ k ≤ -6, 23 ≤ l ≤ -23

Structure Solution and Refinement

Structure solution program	SHELXD (Uson & Sheldrick, 1999)
Primary solution method	Direct methods
Secondary solution method	Difference Fourier map
Structure refinement program	SHELXL-2018/3 (Sheldrick, 2018)
Refinement method	Full matrix least-squares on F^2
Data / restraints / parameters	1740 / 488 / 237
Treatment of hydrogen atoms	Riding
Goodness-of-fit on F^2	1.236
Final R indices [I>2s(I)]	R1 = 0.1345, *w*R2 = 0.3289
R indices (all data)	R1 = 0.1962, *w*R2 = 0.3673
Type of weighting scheme used	Sigma

Weighting scheme used	$w=1/s^2(Fo^2)$
Max shift/error	0.000
Average shift/error	0.000
Largest diff. peak and hole	0.15 and -0.12 e.Å^{-3}

Special Refinement Details

Refinement of F^2 against ALL reflections. The weighted R-factor (wR) and goodness of fit (S) are based on F-^{2}, conventional R-factors (R) are based on F, with F set to zero for negative F^2. The threshold expression of $F^2 > 2s(F^2)$ is used only for calculating R-factors(gt) etc. and is not relevant to the choice of reflections for refinement. R-factors based on F^2 are statistically about twice as large as those based on F, and R-factors based on ALL data will be even larger.

All esds (except the esd in the dihedral angle between two l.s. planes) are estimated using the full covariance matrix. The cell esds are taken into account individually in the estimation of esds in distances, angles and torsion angles; correlations between esds in cell parameters are only used when they are defined by crystal symmetry. An approximate (isotropic) treatment of cell esds is used for estimating esds involving l.s. planes.

4.9 8-hydroxy-8-(hydroxymethyl)-4,4,11b-trimethyldodecahydro-6a,9-methanocyclohepta[a]naphthalen-3(2H)-one (SI-9, CCDC 2246155).

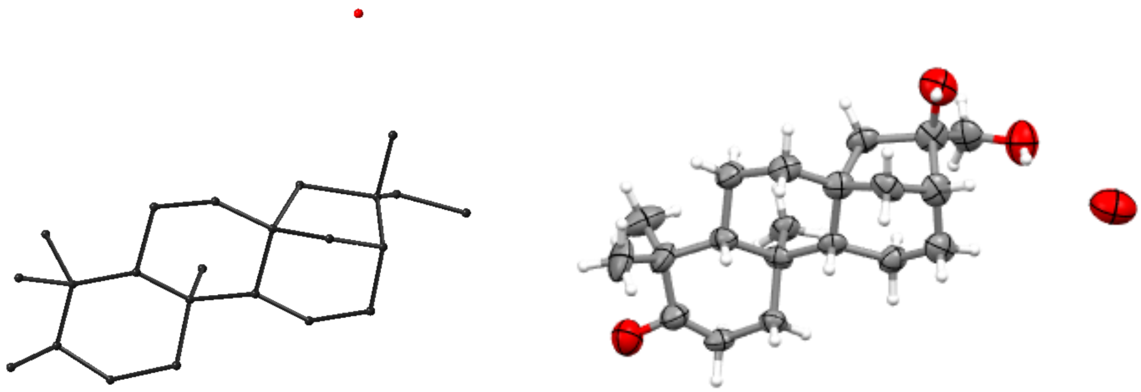

Initial direct methods solution of **SI-9** (left) and ORTEP diagram of refined **SI-9** (right). Thermal ellipsoids shown as shaded octants at 30% probability.

Crystal data and structure refinement for SI-9.

Empirical formula	$C_{40}H_{64}O_7$
Formula weight	656.91

Data Collection

Type of instrument	Talos Arctica F200C
Wavelength	0.0215 Å
Data collection temperature	80(4) K
Unit cell dimensions	a = 13.5600(10)
	b = 6.410(2)
	c = 21.180(4)
	β = 100.25
Volume	1811.6(7)

Z	2
Crystal system	Monoclinic
Space group	C2
Density (calculated)	1.204 Mg/m^3
F(000)	176
Measured reflections	1618
Reflections with I > 2σ(*I*)	962
Resolution	1.0 Å
Completeness	81.9%
Index ranges	13 ≤ h ≤ -13, 6 ≤ k ≤ -6, 20 ≤ l ≤ -20

Structure Solution and Refinement

Structure solution program	SHELXD (Uson & Sheldrick, 1999)
Primary solution method	Direct methods
Secondary solution method	Difference Fourier map
Structure refinement program	SHELXL-2018/3 (Sheldrick, 2018)
Refinement method	Full matrix least-squares on F^2
Data / restraints / parameters	1618 / 371 / 219
Treatment of hydrogen atoms	Riding
Goodness-of-fit on F^2	1.333
Final R indices [I>2s(I)]	R1 = 0.1460, *w*R2 = 0.3008
R indices (all data)	R1 = 0.2054, *w*R2 = 0.3381

Type of weighting scheme used	Sigma
Weighting scheme used	$w=1/s^2(Fo^2)$
Max shift/error	0.000
Average shift/error	0.000
Largest diff. peak and hole	0.27 and -0.32 e.Å^{-3}

Special Refinement Details

Refinement of F^2 against ALL reflections. The weighted R-factor (wR) and goodness of fit (S) are based on F-^{2}, conventional R-factors (R) are based on F, with F set to zero for negative F^2. The threshold expression of $F^2 > 2s(F^2)$ is used only for calculating R-factors(gt) etc. and is not relevant to the choice of reflections for refinement. R-factors based on F^2 are statistically about twice as large as those based on F, and R-factors based on ALL data will be even larger.

All esds (except the esd in the dihedral angle between two l.s. planes) are estimated using the full covariance matrix. The cell esds are taken into account individually in the estimation of esds in distances, angles and torsion angles; correlations between esds in cell parameters are only used when they are defined by crystal symmetry. An approximate (isotropic) treatment of cell esds is used for estimating esds involving l.s. planes.

4.10 6-heptanoyl-4-methoxy-2H-pyran-2-one (SI-10, CCDC 2246161).

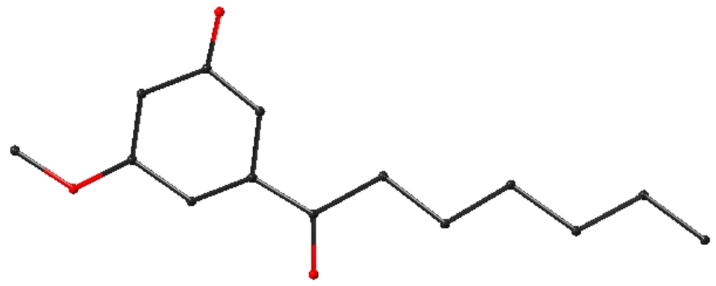

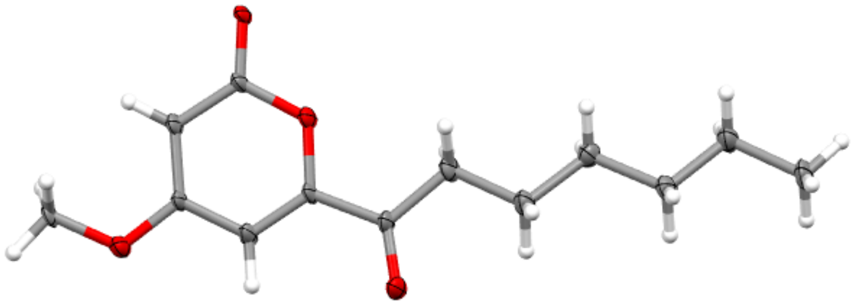

Initial direct methods solution of **SI-10** (top) and ORTEP diagram of refined **SI-10** (bottom). Thermal ellipsoids shown as shaded octants at 30% probability.

Crystal data and structure refinement for SI-10.

Empirical formula	$C_{13}H_{18}O_4$
Formula weight	238.27

Data Collection

Type of instrument	Talos Arctica F200C
Wavelength	0.0215 Å
Data collection temperature	80(4) K
Unit cell dimensions	a = 5.2300(10)
	b = 8.770(2)

	c = 15.060(4)□
	α□= 105.47
	β□= 91.63
	γ□= 107.43
Volume	630.7(3)
Z	2
Crystal system	Triclinic
Space group	P-1
Density (calculated)	1.255 Mg/m^3
F(000)	12
Measured reflections	1840
Reflections with I > 2σ(*I*)	1177
Resolution	0.85 Å
Completeness	86.9%
Index ranges	6 ≤ h ≤ -6, 10 ≤ k ≤ -10, 17 ≤ l ≤ -17

Structure Solution and Refinement

Structure solution program	SHELXT (Uson & Sheldrick, 1999)
Primary solution method	Direct methods
Secondary solution method	Difference Fourier map
Structure refinement program	SHELXL-2018/3 (Sheldrick, 2018)
Refinement method	Full matrix least-squares on F^2

Data / restraints / parameters	1840 / 254 / 155
Treatment of hydrogen atoms	Riding
Goodness-of-fit on F^2	1.550
Final R indices [I>2s(I)]	R1 = 0.1899, *w*R2 = 0.4455
R indices (all data)	R1 = 0.2282, *w*R2 = 0.4746
Type of weighting scheme used	Sigma
Weighting scheme used	$w=1/s^2(Fo^2)$
Max shift/error	0.010
Average shift/error	0.000
Largest diff. peak and hole	0.25 and -0.25 e.Å^{-3}

Special Refinement Details

Refinement of F^2 against ALL reflections. The weighted R-factor (wR) and goodness of fit (S) are based on F-^{2}, conventional R-factors (R) are based on F, with F set to zero for negative F^2. The threshold expression of $F^2 > 2s(F^2)$ is used only for calculating R-factors(gt) etc. and is not relevant to the choice of reflections for refinement. R-factors based on F^2 are statistically about twice as large as those based on F, and R-factors based on ALL data will be even larger.

All esds (except the esd in the dihedral angle between two l.s. planes) are estimated using the full covariance matrix. The cell esds are taken into account individually in the estimation of esds in distances, angles and torsion angles; correlations between esds in cell parameters are only used when they are defined by crystal symmetry. An approximate (isotropic) treatment of cell esds is used for estimating esds involving l.s. planes.

4.11 1,8-dihydroxy-3-methylanthracene-9,10-dione (SI-11, CCDC 2246157).

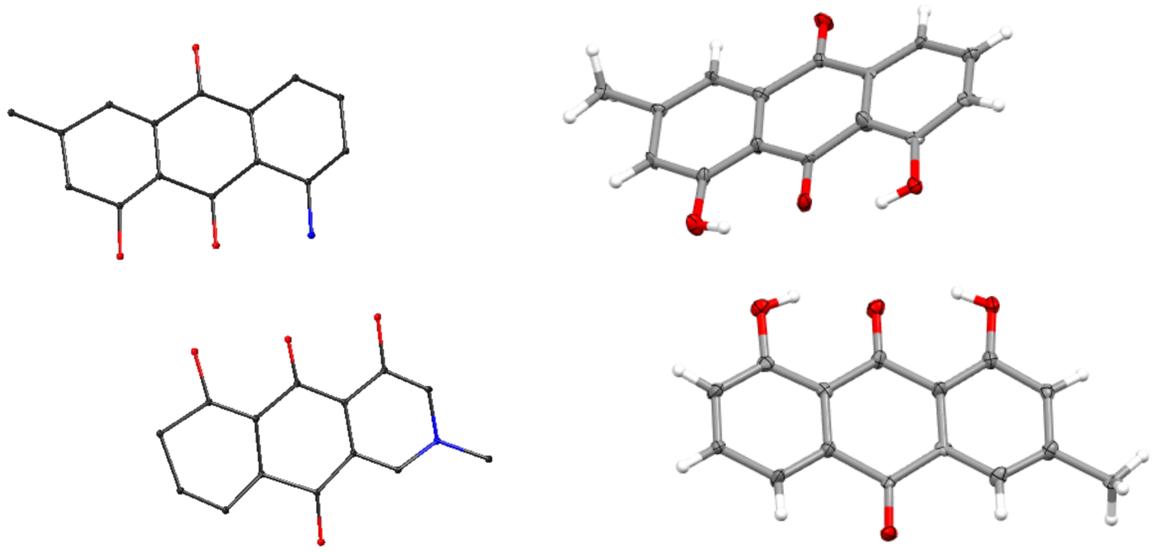

Initial direct methods solution of **SI-11** (left) and ORTEP diagram of refined **SI-11** (right). Thermal ellipsoids shown as shaded octants at 30% probability.

Crystal data and structure refinement for SI-11.

Empirical formula	$C_{15}H_{9.96}O_{3.96}$
Formula weight	253.55

Data Collection

Type of instrument	Talos Arctica F200C
Wavelength	0.0215 Å
Data collection temperature	80(4) K
Unit cell dimensions	a = 3.9200(10)
	b = 23.250(2)
	c = 24.730(4)□

Volume	2253.9(7)
Z	8
Crystal system	Orthorhombic
Space group	$P2_12_12_1$
Density (calculated)	1.494 Mg/m^3
F(000)	12
Measured reflections	3594
Reflections with $I > 2\sigma(I)$	3054
Resolution	0.85 Å
Completeness	90.8%
Index ranges	4 ≤ h ≤ -4, 25 ≤ k ≤ -26, 28 ≤ l ≤ -29

Structure Solution and Refinement

Structure solution program	SHELXT (Uson & Sheldrick, 1999)
Primary solution method	Direct methods
Secondary solution method	Difference Fourier map
Structure refinement program	SHELXL-2018/3 (Sheldrick, 2018)
Refinement method	Full matrix least-squares on F^2
Data / restraints / parameters	3594 / 676 / 349
Treatment of hydrogen atoms	Riding
Goodness-of-fit on F^2	1.380
Final R indices [I>2s(I)]	R1 = 0.1590, *w*R2 = 0.3902

R indices (all data)	R1 = 0.1687, *w*R2 = 0.4003
Type of weighting scheme used	Sigma
Weighting scheme used	$w=1/s^2(Fo^2)$
Max shift/error	0.032
Average shift/error	0.000
Largest diff. peak and hole	0.34 and -0.26 e.Å^{-3}

Special Refinement Details

Refinement of F^2 against ALL reflections. The weighted R-factor (wR) and goodness of fit (S) are based on F^{-2}, conventional R-factors (R) are based on F, with F set to zero for negative F^2. The threshold expression of $F^2 > 2s(F^2)$ is used only for calculating R-factors(gt) etc. and is not relevant to the choice of reflections for refinement. R-factors based on F^2 are statistically about twice as large as those based on F, and R-factors based on ALL data will be even larger.

All esds (except the esd in the dihedral angle between two l.s. planes) are estimated using the full covariance matrix. The cell esds are taken into account individually in the estimation of esds in distances, angles and torsion angles; correlations between esds in cell parameters are only used when they are defined by crystal symmetry. An approximate (isotropic) treatment of cell esds is used for estimating esds involving l.s. planes.

4.12 1-hydroxy-3-methylanthracene-9,10-dione (SI-12, CCDC 2246159).

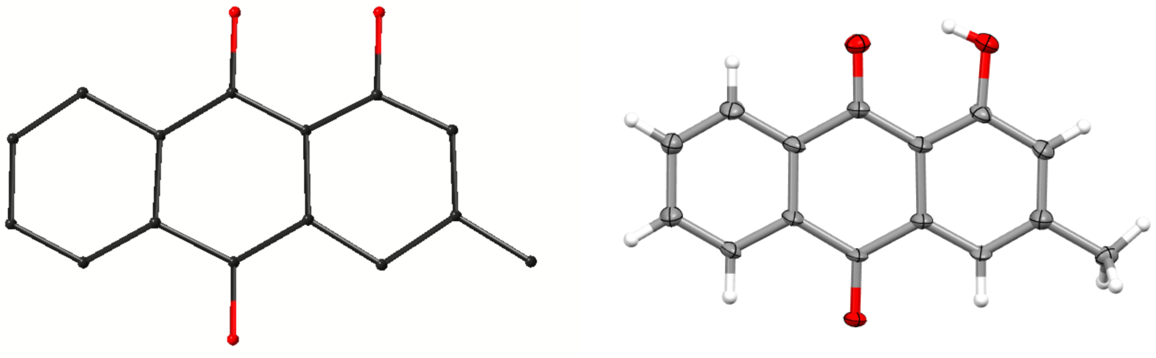

Initial direct methods solution of **SI-12** (left) and ORTEP diagram of refined **SI-12** (right). Thermal ellipsoids shown as shaded octants at 30% probability.

Crystal data and structure refinement for SI-12.

Empirical formula	$C_{15}H_{10}O_3$
Formula weight	238.23

Data Collection

Type of instrument	Talos Arctica F200C
Wavelength	0.0215 Å
Data collection temperature	80(4) K
Unit cell dimensions	a = 3.8300(10)
	b = 12.760(2)
	c = 22.210(4)□
Volume	1084.9(4)
Z	4
Crystal system	Monoclinic
Space group	$P2_1/c$

Density (calculated)	1.459 Mg/m^3
F(000)	12
Measured reflections	1305
Reflections with I > 2σ(*I*)	879
Resolution	0.90 Å
Completeness	82.6%
Index ranges	4 ≤ h ≤ -4, 13 ≤ k ≤ -13, 22 ≤ l ≤ -22

Structure Solution and Refinement

Structure solution program	SHELXT (Uson & Sheldrick, 1999)
Primary solution method	Direct methods
Secondary solution method	Difference Fourier map
Structure refinement program	SHELXL-2018/3 (Sheldrick, 2018)
Refinement method	Full matrix least-squares on F^2
Data / restraints / parameters	1306 / 270 / 165
Treatment of hydrogen atoms	Riding
Goodness-of-fit on F^2	1.657
Final R indices [I>2s(I)]	R1 = 0.1850, *w*R2 = 0.4511
R indices (all data)	R1 = 0.2208, *w*R2 = 0.4827
Type of weighting scheme used	Sigma
Weighting scheme used	w=1/s^2(Fo^2)
Max shift/error	0.009

Average shift/error	0.000
Largest diff. peak and hole	0.20 and -0.16 e.Å^{-3}

Special Refinement Details

Refinement of F^2 against ALL reflections. The weighted R-factor (wR) and goodness of fit (S) are based on F-^{2}, conventional R-factors (R) are based on F, with F set to zero for negative F^2. The threshold expression of $F^2 > 2s(F^2)$ is used only for calculating R-factors(gt) etc. and is not relevant to the choice of reflections for refinement. R-factors based on F^2 are statistically about twice as large as those based on F, and R-factors based on ALL data will be even larger.

All esds (except the esd in the dihedral angle between two l.s. planes) are estimated using the full covariance matrix. The cell esds are taken into account individually in the estimation of esds in distances, angles and torsion angles; correlations between esds in cell parameters are only used when they are defined by crystal symmetry. An approximate (isotropic) treatment of cell esds is used for estimating esds involving l.s. planes.

4.13 5,5,13,13-tetramethyl-3,7,11,15-tetraoxa-1,9(1,4)-dibenzenacyclohexadecaphane-2,8,10,16-tetraone (**SI-13, CCDC 2246160).**

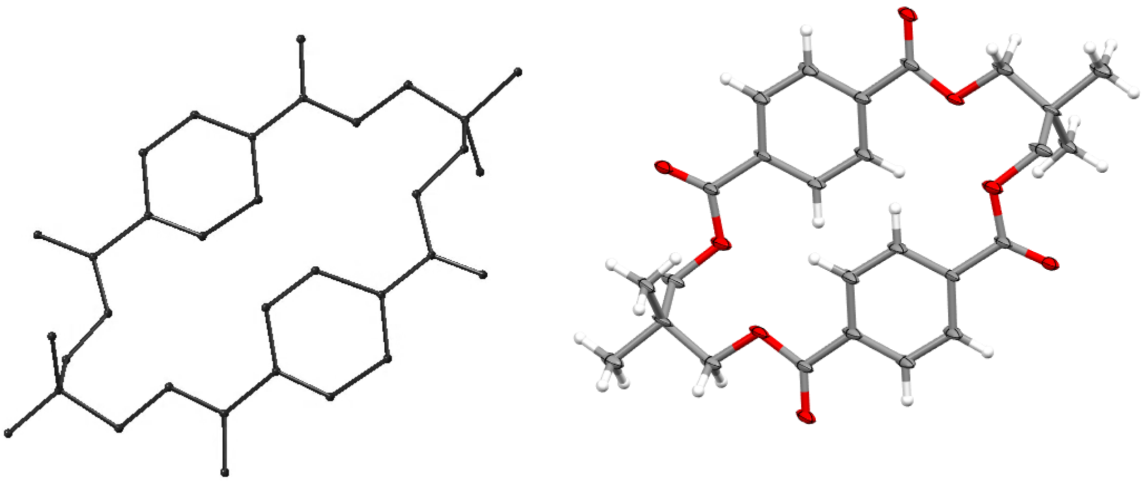

Initial direct methods solution of **SI-13** (left) and ORTEP diagram of refined **SI-13** (right). Thermal ellipsoids shown as shaded octants at 30% probability.

Crystal data and structure refinement for SI-13.

Empirical formula	$C_{26}H_{24}O_8$
Formula weight	464.45

Data Collection

Type of instrument	Talos Arctica F200C
Wavelength	0.0215 Å
Data collection temperature	80(4) K
Unit cell dimensions	a = 6.3200(10)
	b = 9.140(2)
	c = 10.150(4)
	α = 102.44
	β = 94.29

	γ = 91.34
Volume	570.5(3)
Z	1
Crystal system	Triclinic
Space group	P-1
Density (calculated)	1.352 Mg/m^3
F(000)	10
Measured reflections	1337
Reflections with I > $2\sigma(I)$	961
Resolution	0.90 Å
Completeness	80.6%
Index ranges	$6 \le h \le -6$, $9 \le k \le -9$, $11 \le l \le -11$

Structure Solution and Refinement

Structure solution program	SHELXT (Uson & Sheldrick, 1999)
Primary solution method	Direct methods
Secondary solution method	Difference Fourier map
Structure refinement program	SHELXL-2018/3 (Sheldrick, 2018)
Refinement method	Full matrix least-squares on F^2
Data / restraints / parameters	1337 / 219 / 155
Treatment of hydrogen atoms	Riding
Goodness-of-fit on F^2	1.919

Final R indices [I>2s(I)]	R1 = 0.2081, *w*R2 = 0.4907
R indices (all data)	R1 = 0.2409, *w*R2 = 0.5206
Type of weighting scheme used	Sigma
Weighting scheme used	$w=1/s^2(Fo^2)$
Max shift/error	0.043
Average shift/error	0.000
Largest diff. peak and hole	0.21 and -0.28 e.Å^{-3}

Special Refinement Details

Refinement of F^2 against ALL reflections. The weighted R-factor (wR) and goodness of fit (S) are based on $F\text{-}^2$, conventional R-factors (R) are based on F, with F set to zero for negative F^2. The threshold expression of $F^2 > 2s(F^2)$ is used only for calculating R-factors(gt) etc. and is not relevant to the choice of reflections for refinement. R-factors based on F^2 are statistically about twice as large as those based on F, and R-factors based on ALL data will be even larger.

All esds (except the esd in the dihedral angle between two l.s. planes) are estimated using the full covariance matrix. The cell esds are taken into account individually in the estimation of esds in distances, angles and torsion angles; correlations between esds in cell parameters are only used when they are defined by crystal symmetry. An approximate (isotropic) treatment of cell esds is used for estimating esds involving l.s. planes.

4.14 1,8-dihydroxy-3-methylanthracene-9,10-dione, 1-hydroxy-3-methylanthracene-9,10-dione (SI-14, CCDC 2246164).

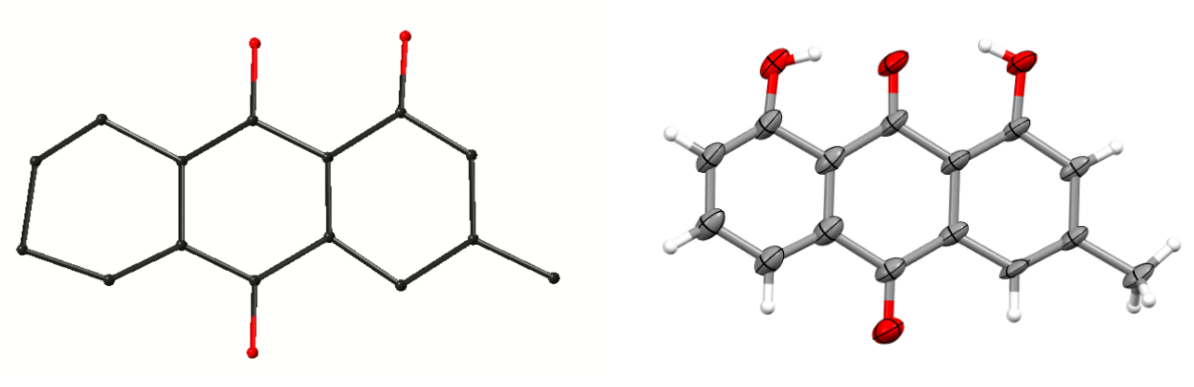

Initial direct methods solution of **SI-14** (left) and ORTEP diagram of refined **SI-14** (right). Thermal ellipsoids shown as shaded octants at 30% probability.

Crystal data and structure refinement for SI-14.

Empirical formula	$C_{15}H_{9.24}O_{3.24}$
Formula weight	241.30

Data Collection

Type of instrument	Talos Arctica F200C
Wavelength	0.0215 Å
Data collection temperature	80(4) K
Unit cell dimensions	a = 8.2200(10)
	b = 3.830(2)
	c = 34.870(4)
	β = 92.81
Volume	1096.5(6)
Z	4

Crystal system	Monoclinic
Space group	$P2_1/c$
Density (calculated)	1.462 Mg/m^3
F(000)	10
Measured reflections	1696
Reflections with $I > 2\sigma(I)$	893
Resolution	0.85 Å
Completeness	86.6%
Index ranges	9 ≤ h ≤ -9, 4 ≤ k ≤ -4, 40 ≤ l ≤ -40

Structure Solution and Refinement

Structure solution program	SHELXT (Uson & Sheldrick, 1999)
Primary solution method	Direct methods
Secondary solution method	Difference Fourier map
Structure refinement program	SHELXL-2018/3 (Sheldrick, 2018)
Refinement method	Full matrix least-squares on F^2
Data / restraints / parameters	1696 / 340 / 176
Treatment of hydrogen atoms	Riding
Goodness-of-fit on F^2	1.612
Final R indices [I>2s(I)]	R1 = 0.2046, *w*R2 = 0.5103
R indices (all data)	R1 = 0.2635, *w*R2 = 0.5416
Type of weighting scheme used	Sigma

Weighting scheme used	$w=1/s^2(Fo^2)$
Max shift/error	0.004
Average shift/error	0.000
Largest diff. peak and hole	0.20 and -0.18 e.Å^{-3}

Special Refinement Details

Refinement of F^2 against ALL reflections. The weighted R-factor (wR) and goodness of fit (S) are based on $F\text{-}^2$, conventional R-factors (R) are based on F, with F set to zero for negative F^2. The threshold expression of $F^2 > 2s(F^2)$ is used only for calculating R-factors(gt) etc. and is not relevant to the choice of reflections for refinement. R-factors based on F^2 are statistically about twice as large as those based on F, and R-factors based on ALL data will be even larger.

All esds (except the esd in the dihedral angle between two l.s. planes) are estimated using the full covariance matrix. The cell esds are taken into account individually in the estimation of esds in distances, angles and torsion angles; correlations between esds in cell parameters are only used when they are defined by crystal symmetry. An approximate (isotropic) treatment of cell esds is used for estimating esds involving l.s. planes.

4.15 1,3,8-trihydroxy-6-methylanthracene-9,10- (SI-15, CCDC 2246163).

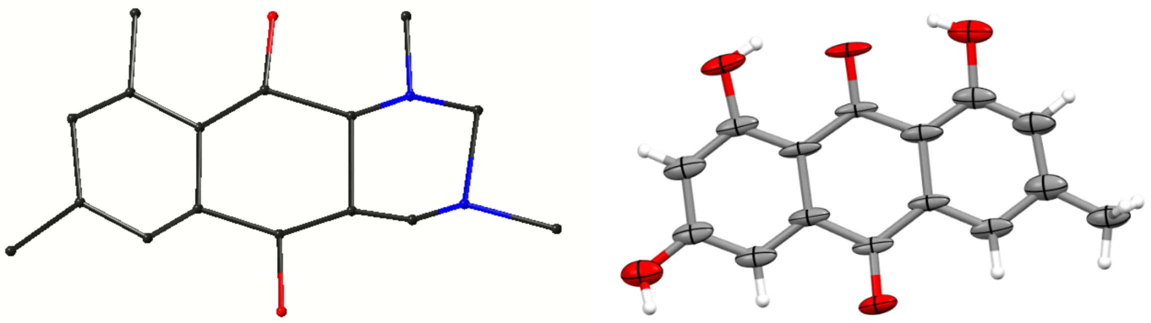

Initial direct methods solution of **SI-15** (left) and ORTEP diagram of refined **SI-15** (right). Thermal ellipsoids shown as shaded octants at 30% probability.

Crystal data and structure refinement for SI-15.

Empirical formula	$C_{15}H_{10}O_5$
Formula weight	270.23

Data Collection

Type of instrument	Talos Arctica F200C
Wavelength	0.0215 Å
Data collection temperature	80(4) K
Unit cell dimensions	a = 16.7200(10)
	b = 3.720(2)
	c = 19.530(4)
	β = 95.24
Volume	1209.7(7)
Z	4

Crystal system	Monoclinic
Space group	$P2_1/n$
Density (calculated)	1.484 Mg/m^3
F(000)	10
Measured reflections	1786
Reflections with $I > 2\sigma(I)$	1086
Resolution	0.90 Å
Completeness	82.1%
Index ranges	19 ≤ h ≤ -19, 4 ≤ k ≤ -4, 23 ≤ l ≤ -23

Structure Solution and Refinement

Structure solution program	SHELXT (Uson & Sheldrick, 1999)
Primary solution method	Direct methods
Secondary solution method	Difference Fourier map
Structure refinement program	SHELXL-2018/3 (Sheldrick, 2018)
Refinement method	Full matrix least-squares on F^2
Data / restraints / parameters	1786 / 357 / 185
Treatment of hydrogen atoms	Riding
Goodness-of-fit on F^2	1.701
Final R indices [I>2s(I)]	R1 = 0.2355, *w*R2 = 0.5486
R indices (all data)	R1 = 0.2738, *w*R2 = 0.5669
Type of weighting scheme used	Sigma

Weighting scheme used	$w=1/s^2(Fo^2)$
Max shift/error	0.024
Average shift/error	0.000
Largest diff. peak and hole	0.16 and -0.18 e.Å^{-3}

Special Refinement Details

Refinement of F^2 against ALL reflections. The weighted R-factor (wR) and goodness of fit (S) are based on F-^{2}, conventional R-factors (R) are based on F, with F set to zero for negative F^2. The threshold expression of $F^2 > 2s(F^2)$ is used only for calculating R-factors(gt) etc. and is not relevant to the choice of reflections for refinement. R-factors based on F^2 are statistically about twice as large as those based on F, and R-factors based on ALL data will be even larger.

All esds (except the esd in the dihedral angle between two l.s. planes) are estimated using the full covariance matrix. The cell esds are taken into account individually in the estimation of esds in distances, angles and torsion angles; correlations between esds in cell parameters are only used when they are defined by crystal symmetry. An approximate (isotropic) treatment of cell esds is used for estimating esds involving l.s. planes.

4.16 Preliminary solution of (SI-16).

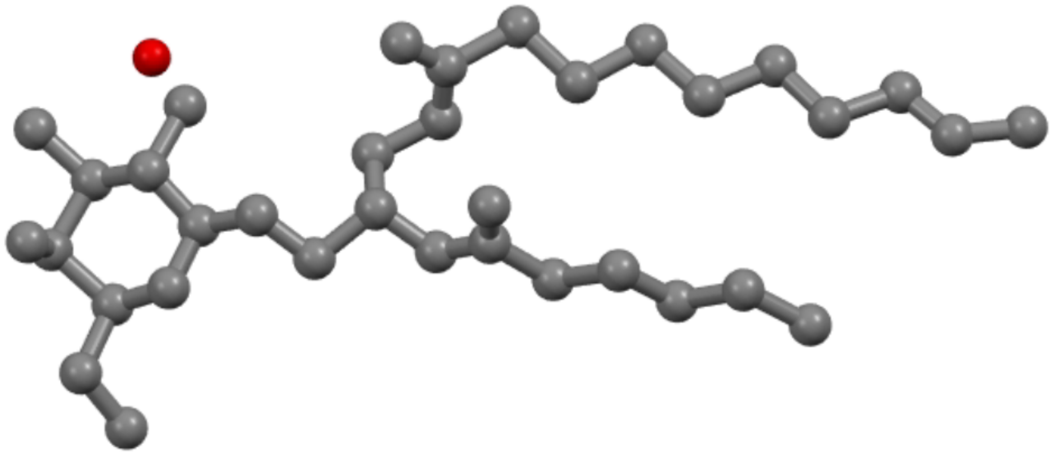

Initial direct methods solution of **SI-16**.

Crystal data and structure refinement for SI-16.

Empirical formula	$C_{15}H_{10}O_5$
Formula weight	270.23

Data Collection

Type of instrument	Talos Arctica F200C
Wavelength	0.0215 Å
Data collection temperature	80(4) K
Unit cell dimensions	a = 8.1500(10)
	b = 5.340(2)
	c = 53.730(4)
	β = 91.76

Volume	2337.3(9)
Z	4
Crystal system	Monoclinic
Space group	$P2_1$
Density (calculated)	1.484 Mg/m^3
F(000)	26
Measured reflections	4314
Reflections with I > $2\sigma(I)$	2142
Resolution	0.90 Å
Completeness	82.1%
Index ranges	8 ≤ h ≤ -8, 5 ≤ k ≤ -5, 49 ≤ l ≤ -50

5. References

1. SERNEC Southeast Regional Network of Expertise and Collections. (accessed 2020-01-02) http://sernecportal.org/portal/.
2. Dettweiler, M.; Melander, R. J.; Porras, G.; Risener, C.; Marquez, L.; Samarakoon, T.; Melander, C.; Quave, C. L. A Clerodane Diterpene from *Callicarpa americana* Resensitizes Methicillin-Resistant *Staphylococcus aureus* to β-Lactam Antibiotics. *ACS Infect. Dis.* **2020**, *6*, 1667– 1673, DOI: 10.1021/acsinfecdis.0c00307
3. Littler, D.; Littler, M. South Pacific reef plants. A divers' guide to the plant life of South Pacific coral reefs. Offshore Graphics. *Inc., Washington, DC* **2003**, 331.
4. Jones, C. G.; Martynowycz, M. W.; Hattne, J.; Fulton, T. J.; Stoltz, B. M.; Rodriguez, J. A.; Nelson, H. M.; Gonen, T. The cryoEM method MicroED as a powerful tool for small molecule structure determination. *ACS Cent. Sci.* **2018**, *4*, 1587−1592.
5. Kabsch, W. Acta Cryst. **2010**, *D66*, 125–132.
6. Kabsch, W. *Acta Cryst.* **2010**, *D66*, 133–144.
7. Hattne, J., *et al. Acta Cryst.* **2015**, *71*, 353–360.
8. Sheldrick, G. M. A short history of SHELX. *Acta Cryst.* **2008**, *A64*, 112–122.
9. Sheldrick, G. M. *Acta Cryst.* **2015**, *A71*, 3–8.
10. Sheldrick, G. M. *Acta Cryst.* **2015**, *C71*, 3–8.
11. Hübschle, C. B.; Sheldrick, G. M.; Dittrich, B. *J. Appl. Cryst.* **2011**, *44*, 1281–1284.
